# Recent Advances in Additively Manufactured Polymeric Structures for Mechanical Energy Absorption

**DOI:** 10.3390/polym18091019

**Published:** 2026-04-23

**Authors:** Alin Bustihan, Ioan Botiz

**Affiliations:** 1 Department of Physics of Condensed Matter and Advanced Technologies, Faculty of Physics, Babeș-Bolyai University, 400084 Cluj-Napoca, Romania; alin.bustihan@ubbcluj.ro; 2 Interdisciplinary Research Institute on Bio-Nano-Sciences, Babeș-Bolyai University, 400271 Cluj-Napoca, Romania

**Keywords:** additive manufacturing, 3D-printed polymeric structures, lattice structures, metamaterials, mechanical properties, energy-absorbing applications

## Abstract

Additive manufacturing has emerged as a powerful approach for producing architected materials with tailored mechanical properties and enhanced energy absorption capabilities. By enabling precise control over geometry, relative density, and hierarchical topology, additive manufacturing facilitates the design of lightweight cellular structures with superior crashworthiness compared to conventional energy-absorbing materials. This review provides a comprehensive overview of recent advances in additively manufactured energy-absorbing structures, with particular emphasis on the interplay between structural architecture, fabrication technologies, and mechanical performance. Key additive manufacturing processes, including fused deposition modeling, stereolithography, selective laser sintering, and multi-jet fusion, are evaluated in terms of their fabrication capabilities, material compatibility, and inherent limitations. Special attention is given to the mechanical behavior of representative architectures, including two-dimensional cellular structures, three-dimensional lattice geometries, sandwich systems, and emerging four-dimensional programmable materials. Depending on topology and material system, additively manufactured lattices can achieve specific energy absorption values exceeding 20–40 J g^−1^, significantly outperforming many conventional foams. Finally, current challenges, such as process-induced defects, anisotropic mechanical behavior, and the lack of standardized testing methodologies, are discussed, along with future research directions, including multi-material printing, functionally graded architectures, and adaptive metamaterials for next-generation impact mitigation systems.

## 1. Introduction

### 1.1. Motivation and Background

The increasing demand for lightweight, high-performance structures capable of dissipating mechanical energy has driven significant innovation in materials science and engineering over the past decade. Traditional energy-absorbing materials, such as metallic foams and conventional polymer foams, while effective, often suffer from limitations including high density, limited design flexibility, and inability to tailor mechanical properties for specific loading conditions [1,2]. The emergence of additive manufacturing (AM) technologies has fundamentally transformed the landscape of energy absorption research by enabling the fabrication of complex, geometrically optimized structures that were previously impossible to manufacture using conventional methods [3,4].

Polymeric materials constitute a class of soft matter [5,6,7,8] that has been extensively employed in the fabrication of miniaturized surface relief structures and nanoscale features via both top-down and bottom-up approaches [9,10,11,12,13,14]. Their broad applicability spans multiple domains of materials science, including biomedical engineering, electronics, and energy systems. Owing to their favorable strength-to-weight ratios, high design flexibility, and cost-effectiveness, polymeric materials have also emerged as particularly promising candidates for energy absorption applications [15]. When integrated with AM technologies, polymers facilitate the fabrication of architected cellular structures with precisely tailored geometries, relative densities, and mechanical responses [16,17]. Such 3D-printed polymeric architectures can be systematically engineered to exhibit specific deformation mechanisms, such as progressive buckling, plastic hinge formation, and controlled densification, that enhance energy dissipation while minimizing peak forces transmitted to protected systems or occupants [17,18].

Recent advances in computational design, topology optimization, and multi-material printing have significantly expanded the design space for energy-absorbing structures [19,20]. Researchers have demonstrated that by manipulating unit cell geometry, relative density, and material distribution, it is possible to achieve energy absorption performance that rivals or surpasses that of traditional metallic structures while maintaining substantially lower mass [21]. Furthermore, the ability to rapidly prototype and iterate designs through AM has accelerated the development cycle for energy-absorbing components, enabling more efficient translation from concept to application [22]. Additionally, the integration of bio-inspired design principles with AM technologies has opened new avenues for innovation in energy absorption [23]. Natural structures such as trabecular bone, wood cellular architecture, and crustacean exoskeletons have evolved over millions of years to efficiently absorb and dissipate mechanical energy [1]. By mimicking these hierarchical, functionally graded architectures through 3D printing, researchers have achieved remarkable improvements in specific energy absorption (SEA) and crush load efficiency (CLE) compared to uniform structures [24,25].

Despite these advances, significant challenges remain in translating laboratory-scale demonstrations to industrial applications. Key issues include the impact of manufacturing defects on mechanical performance, the rate-dependent behavior of polymeric materials under dynamic loading, and the need for standardized testing protocols that enable meaningful comparisons across studies [26,27]. Additionally, the environmental sustainability of 3D-printed energy absorbers, including material recyclability and reusability after impact events, has emerged as a critical consideration for practical implementation [28,29].

### 1.2. Additive Manufacturing Technologies for Energy Absorption

The selection of AM technology critically influences the mechanical performance, geometric fidelity, and energy absorption behavior of 3D-printed polymeric structures. Among available techniques, fused deposition modeling (FDM), stereolithography (SLA), and selective laser sintering (SLS) are the most extensively employed for energy absorption applications, each offering distinct advantages and limitations.

FDM remains the most widely utilized method due to its accessibility, material versatility, and cost-effectiveness [30,31,32]. It employs layer-by-layer extrusion of thermoplastic filaments, including PLA, ABS, PETG, TPU, and PEBA [15,33,34]. However, its mechanical performance is highly dependent on process parameters and is inherently anisotropic due to interlayer bonding characteristics [1,35,36,37]. While this anisotropy can be exploited to tailor directional energy absorption [38], interlayer voids and weak interfaces may act as crack initiation sites, reducing structural integrity [39]. Notably, FDM enables the use of flexible polymers, such as TPU and PEBA, which exhibit high resilience and reusability under impact loading [15,28].

In contrast, SLA and related vat photopolymerization techniques provide superior resolution and surface quality, enabling the fabrication of intricate lattice geometries with fine feature control [40,41]. These structures typically exhibit more isotropic mechanical properties due to improved interlayer bonding [42], making them suitable for complex architectures such as TPMS lattices [43,44]. Despite their favorable geometric precision and demonstrated energy absorption performance [41], SLA materials are generally more brittle and less suitable for applications requiring large deformation or repeated use [40,42].

SLS utilizes laser-induced fusion of polymer powders to produce support-free, geometrically complex overhanging geometries and hollow structures with relatively uniform mechanical properties and enhanced strength [40]. It supports high-performance materials such as PA and PA-CF composites, with reported specific energy absorption exceeding 13 J/g for TPMS structures [38]. Nevertheless, high equipment costs and surface roughness associated with the sintering process remain notable limitations.

Emerging AM technologies, including multi-jet fusion (MJF), continuous liquid interface production (CLIP), and multi-material printing, further expand design possibilities by enabling improved process efficiency, functional grading, and bio-inspired architectures [37,40]. Additionally, 4D printing introduces stimuli-responsive materials capable of shape recovery, offering new opportunities for reusable energy-absorbing systems [37].

### 1.3. Architectural Classifications

The geometric architecture of 3D-printed structures governs their deformation mechanisms and energy absorption performance. Architected energy absorbers can be broadly classified into three categories: (i) 2D cellular structures, (ii) 3D lattice architectures, and (iii) advanced graded, hierarchical, and functional systems.

2D cellular structures consist of periodic planar unit cells extruded in the thickness direction, forming prismatic geometries with distinct in-plane and out-of-plane responses [1]. Hexagonal honeycombs remain the most studied configuration, dissipating energy through progressive wall buckling, plastic hinge formation, and densification [20,43,44]. Their performance depends strongly on relative density and geometric parameters [45]. Variants such as square, triangular, and re-entrant (auxetic) cells provide tunable stiffness and deformation modes, with auxetic designs exhibiting enhanced indentation resistance and more uniform stress distribution [2,4,19,36]. Functional strategies, including density grading and hierarchical design, further improve crush efficiency and stabilize plateau stress by activating multiple deformation mechanisms [28,29].

3D lattice architectures extend cellular design into three dimensions, generally offering higher specific energy absorption and tunable anisotropy [16,18]. These can be categorized into: (1) beam-based lattices (e.g., BCC, FCC, octet), which deform via strut bending or stretching [16]; (2) plate-based lattices, which exhibit shell buckling and folding with higher stiffness-to-weight ratios [40,41]; and (3) triply periodic minimal surface (TPMS) structures, characterized by smooth, continuous geometries that minimize stress concentrations and promote stable deformation [3,17,22]. TPMS lattices, particularly gyroid-based designs, have demonstrated superior energy absorption compared to conventional honeycombs and strut lattices, with reported SEA values exceeding 13 J/g [3,23,38]. Their deformation typically involves progressive layer collapse and shear band formation, enabling stable energy dissipation [35].

Advanced architectures include functionally graded, hierarchical, auxetic, and stimuli-responsive systems that further enhance performance and functionality. Graded lattices optimize stress distribution and delay densification, improving energy absorption efficiency [29]. Hierarchical designs, inspired by natural materials, activate multi-scale deformation mechanisms to extend the energy absorption plateau [29,30]. 3D auxetic structures exhibit improved load distribution and recoverability, particularly when fabricated from flexible polymers [2,4,15]. Additionally, 4D-printed and multi-material systems introduce shape memory and adaptive behavior, enabling reversible energy absorption and structural reconfiguration [37,46]. Recent advances in topology optimization and hybrid reinforcement strategies have also produced non-intuitive geometries with superior performance compared to traditional periodic designs [26,39].

A systematic understanding of energy absorption requires the classification of dominant failure modes and their direct correlation with architectural topology. In general, bending-dominated lattices (e.g., honeycombs, diamond TPMS) fail through progressive elastic buckling and plastic hinging. This layer-by-layer, localized collapse results in a long and stable stress plateau, which is ideal for sustained energy dissipation. In contrast, stretching-dominated lattices (e.g., octet-truss) exhibit higher initial stiffness and peak stress but often fail through catastrophic strut fracture or brittle collapse, leading to abrupt load drops. Furthermore, the AM process introduces additional, process-specific failure mechanisms, most notably interlayer delamination and shear band formation along print paths. These effects can prematurely truncate the energy absorption plateau if structural anisotropy is not properly controlled.

### 1.4. Applications and Industrial Relevance

The combination of low density, tunable mechanical response, and geometric complexity afforded by additively manufactured polymeric energy absorbers has enabled their adoption across multiple industrial sectors. Translating these systems into practical applications requires consideration of domain-specific performance requirements, including impact conditions, weight constraints, and durability.

Automotive and transportation represent key application areas, where crashworthiness components (e.g., bumper cores, side-impact structures, and crumple zones) must dissipate impact energy while limiting transmitted forces. TPMS lattices, particularly gyroid geometries, have demonstrated suitability for crash energy management [3,26,38]. Functionally graded designs enable progressive crushing and improved energy absorption under varying impact scenarios [29]. In electric vehicles, lightweight polymer lattices can replace metallic foams, achieving mass reductions of 30–50% while maintaining comparable performance, with additional design flexibility for integration into complex geometries.

Personal protective equipment (PPE), including helmets and body armor, benefits from the ability to tailor lattice architectures to individual users and impact conditions. Flexible polymers such as TPU and PEBA provide a combination of energy absorption, comfort, and breathability [15], while reusable designs capable of recovering post-impact are advantageous for repeated-use scenarios [29].

Aerospace applications require high specific energy absorption under strict weight constraints. Additively manufactured lattice structures enable multifunctional components that combine load-bearing and impact mitigation. Carbon fiber-reinforced TPMS structures have achieved SEA values exceeding 13 J/g, approaching aerospace requirements [38,40]. In contrast, civil infrastructure and safety systems utilize energy-absorbing structures for impact mitigation in barriers, rockfall protection, and seismic applications. Although scalability remains a challenge, hybrid sandwich structures with 3D-printed cores have demonstrated improved energy absorption and reduced weight compared to solid systems [1,17,30].

Packaging and logistics applications exploit lattice structures for lightweight, customizable protection of fragile goods. Directional stiffness can be tailored to anticipated loading conditions, while rapid prototyping enables product-specific designs. Biodegradable polymers such as PLA offer additional environmental benefits for disposable systems [45].

Biomedical applications leverage lattice architectures for implants, prosthetics, and rehabilitation devices, where mechanical compatibility with biological tissues is essential. Additive manufacturing enables patient-specific designs that mimic the energy absorption behavior of natural tissues, improving functionality and comfort.

Despite these advances, several challenges hinder widespread industrial adoption. Standardized testing protocols and performance metrics are required for reliable comparison and certification [40]. Long-term durability under cyclic loading and environmental exposure remains insufficiently characterized, and manufacturing scalability and cost continue to limit large-scale deployment [28]. Addressing these issues is critical for the broader implementation of 3D-printed polymeric energy absorbers in industry.

## 2. Experimental Methods

### 2.1. Parameters Used to Fabricate Structures via FDM

**Figure 1:**
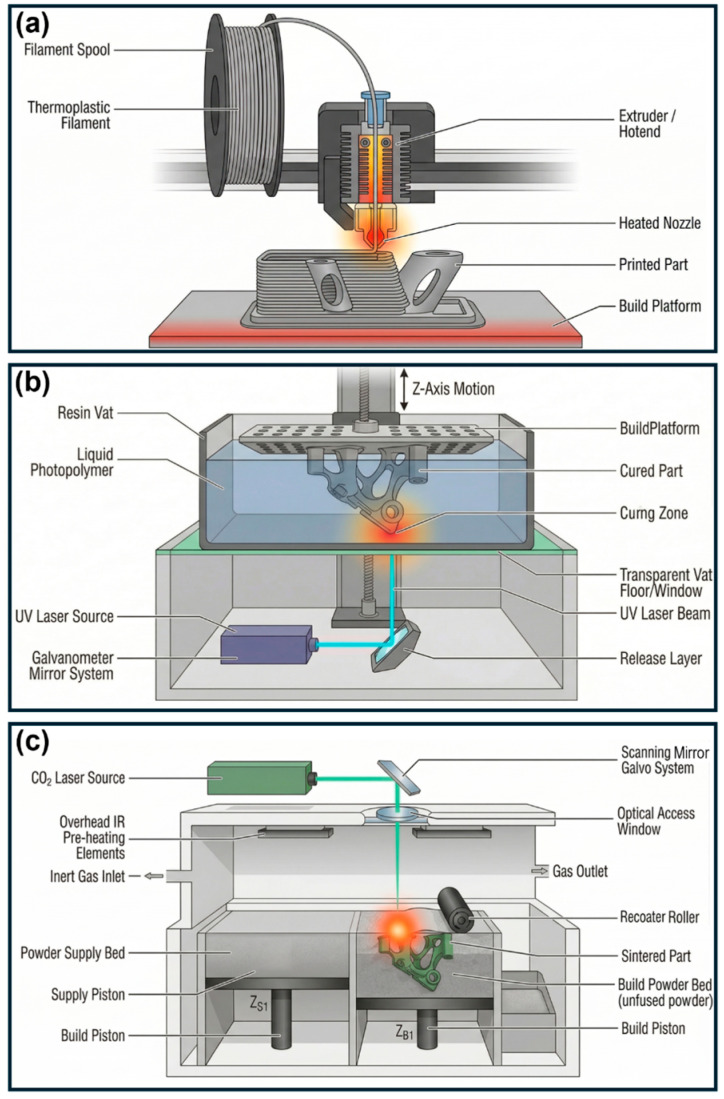
Schematic representation of common additive manufacturing techniques: (**a**) fused deposition modeling (FDM), where a thermoplastic filament is extruded through a heated nozzle; (**b**) vat photopolymerization (SLA/DLP), where a liquid photopolymer is selectively cured by a UV light source; and (**c**) powder bed fusion (SLS/SLM), where a laser selectively sinters or melts powder particles to build the structure layer-by-layer.

FDM is the most widely used technique due to its accessibility, material versatility, and ability to process both rigid and flexible thermoplastics [30,31] (Figure 1a). Common materials include PLA [29,47,48], ABS [48,49], PA [50,51,52], TPU [53,54,55], PETG [45,56] and PEBA [15], with material selection governed by loading conditions. Filaments must be stored under low humidity (<20% RH) to prevent moisture-induced degradation and printing defects [57].

Lattice structures are designed using parametric CAD tools, enabling precise control over unit cell geometry and relative density. Slicing software is typically used to generate G-code, with key parameters including layer height (0.1–0.3 mm), infill density (100% for lattice struts), shell thickness, and support strategy.

Typical nozzle temperatures range from 190 to 270 °C depending on material, with bed temperatures between 40 and 110 °C and print speeds of 20–60 mm/s. Reduced layer height improves interlayer bonding, but at the expense of print time [58]. Due to inherent anisotropy, mechanical properties are strongly influenced by build orientation, with vertical configurations (parallel to the loading direction) exhibiting higher strength but reduced ductility compared to horizontal orientations [1,2]. This structural anisotropy is particularly critical for energy absorption; for instance, interlayer delamination can act as a premature failure mechanism under dynamic loading, significantly reducing the effective energy absorption plateau compared to loading directions that compress the layers together.

FDM-printed specimens undergo support removal and, where required, annealing below the glass transition temperature (e.g., 60–80 °C for PLA, 1–4 h) to relieve residual stresses and enhance interlayer bonding [59]. Surface finishing may be applied when necessary to improve dimensional accuracy, though this is generally less critical for energy absorption testing than for esthetic applications [15,28].

### 2.2. Conditions for Structure Fabrication Using SLA and Digital Light Processing (DLP)

SLA/DLP technologies are employed for the fabrication of high-resolution lattice structures, particularly TPMS geometries, due to their superior surface finish and geometric accuracy [60,61] (Figure 1b). In this case, photopolymer resins (standard, tough, and flexible) are selected based on target mechanical properties and are thoroughly mixed prior to printing to ensure uniform distribution of photoinitiators and additives.

Key parameters include layer thickness (25–100 μm), exposure time (1–10 s), and lift speed. Thinner layers typically improve resolution and surface quality but increase fabrication time. Support structures are typically required for overhanging features and should be designed to minimize contact area with the part to facilitate removal and reduce surface damage. Printed parts are then washed in isopropyl alcohol for 5–10 min to remove uncured resin, followed by UV post-curing (365–405 nm, 15–60 min) to achieve full mechanical properties. Support structures should be carefully removed to preserve fine lattice features [41].

### 2.3. Main Experimental Parameters for SLS and Multi-Jet Fusion (MJF)

SLS/MJF technologies are generally utilized to fabricate support-free, high-performance polymer structures with complex geometries [62,63] (Figure 1c). Common materials for energy absorption applications include PA12, PA11, and PA-CF. Powders are sieved to ensure uniform particle size distribution (40–80 μm) and conditioned in controlled environments to prevent moisture uptake.

Key printing parameters include laser power (15–50 W), scan speed (1000–5000 mm/s), layer thickness (80–150 μm), and bed temperature maintained near the polymer melting point. Energy density needs to be carefully controlled to optimize part density and mechanical performance. Printed structures are then removed from the powder bed and cleaned using compressed air or bead blasting. Additional treatments, such as infiltration or coating, are applied when improved surface finish or mechanical performance is required [38]. A summary of the three printing methods described above is presented in Table 1.

### 2.4. Quality Control and Characterization of Additively Manufactured Structures

Ensuring geometric accuracy and material consistency is critical for achieving reproducible energy absorption results. Accordingly, several quality control procedures are recommended.

Printed specimens should be measured using calipers or coordinate measuring machines (CMM) to ensure that key geometrical parameters, such as cell size, wall thickness, and overall dimensions, match design specifications. Typical tolerances are ±0.1–0.2 mm for FDM and ±0.05–0.1 mm for SLA/SLS. Deviations beyond these ranges may indicate printing defects or suboptimal process parameters [64].

Furthermore, specimens should be weighed using precision balances (±0.001 g) to determine actual mass and calculate relative density, defined as the ratio between lattice and solid material density. Discrepancies from designed values may reveal incomplete material deposition, internal porosity, or inconsistencies in feedstock quality [65,66].

**Figure 2:**
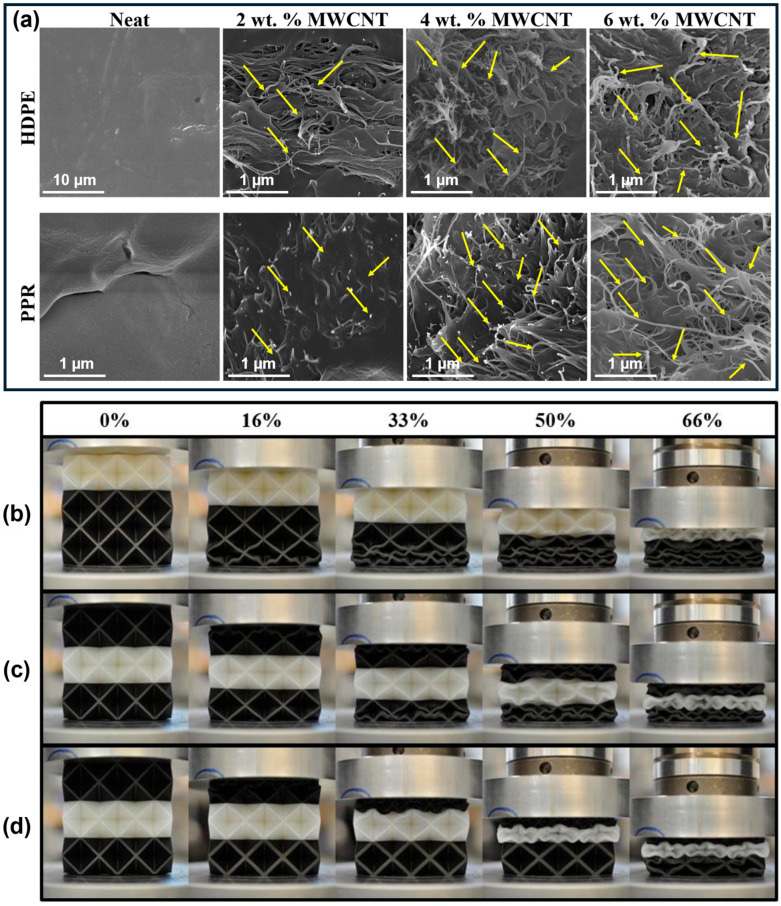
(**a**) SEM micrographs of 3D-printed SC-BCC-FCC structures showing the surface morphology of HDPE, PPR, and their nanocomposites with varying MWCNT contents (arrows indicate the presence of MWCNTs). (**b**–**d**) Quasi-static compression behavior at successive strain intervals for octet lattice structures: (**b**) top nylon configuration, (**c**) middle nylon configuration, and (**d**) middle nylon configuration with cell grading. Reproduced from ref. [81] (**a**) and ref. [52] (**b**–**d**).

Additionally, sample specimens should be visually inspected for macroscopic defects such as warping, layer delamination, incomplete features, or surface irregularities [67]. Scanning electron microscopy (SEM) can further be employed to evaluate surface morphology, interlayer bonding, and microstructural characteristics at higher magnifications [67,68,69] (Figure 2a).

Finally, mechanical properties should be assessed through standardized tensile testing (ASTM D638 [70] or ISO 527 [71]) to determine Young’s modulus, yield strength, ultimate tensile strength, and elongation at break. While these protocols are traditionally intended for bulk materials, they are used here to characterize the polymer properties after printing. This step is critical for providing accurate input parameters for numerical constitutive models, thereby enabling a more precise correlation between finite element simulations and experimental results for complex, anisotropic lattice architectures. Additionally, dynamic mechanical analysis (DMA) may be used to evaluate viscoelastic behavior and temperature-dependent properties [67,72,73].

### 2.5. Testing Protocols

Mechanical characterization of 3D-printed energy-absorbing structures includes quasi-static compression, dynamic impact, and flexural testing, providing complementary insights into deformation mechanisms and energy absorption behavior. The use of standardized protocols ensures comparability across studies and supports model validation. Specifically, by operating within the well-defined testing envelopes detailed in the subsequent sections (e.g., controlled boundary conditions and specific strain-rate regimes), researchers can extract reliable and repeatable force–displacement data. This consistency is essential for defining accurate boundary conditions and validating the predictive capabilities of numerical and finite element models against experimental results.

#### 2.5.1. Quasi-Static Compression Testing

Quasi-static compression testing is employed to evaluate stress–strain response, deformation modes, and energy absorption under controlled loading conditions (Figure 2b–d). Tests are typically conducted using universal testing machines (UTMs) with load capacities of 10–100 kN, equipped with high-accuracy load cells (±0.5%) and displacement measurement systems (±0.01 mm resolution). Compression is applied at constant crosshead speeds of 1–10 mm/min, corresponding to strain rates of approximately 10^−4^ to 10^−2^ s^−1^. Testing is continued until densification (typically 60–80% strain) or machine limits are reached. To ensure repeatability, multiple specimens (*n* = 3–5) are tested for each configuration, enabling statistical analysis of the results [15,74,75].

#### 2.5.2. Dynamic Impact and Low-Velocity Impact Testing

Dynamic testing assesses energy absorption under high strain rate conditions representative of real impact events. Drop-weight impact testing is commonly employed, where a mass is released from a controlled height to generate a defined impact energy. Typical setups include instrumented drop towers with impact masses of 5–50 kg and drop heights of 0.5–5 m, corresponding to impact velocities of 3–10 m/s and strain rates of 10^2^–10^3^ s^−1^. The impactor is equipped with force sensors and accelerometers to capture force–time and acceleration responses during impact [56,76].

#### 2.5.3. Three-Point Bending and Flexural Testing

Three-point bending tests are used to evaluate the flexural response and energy absorption of sandwich structures incorporating 3D-printed cores. Testing is typically conducted according to ASTM C393 [77] or ISO 14125 standards [78]. Specimens are supported over a span length of approximately 10–20 times their thickness and loaded at the midpoint until failure or a predefined deflection. Load–displacement data are used to determine flexural stiffness, peak load, and energy absorption under bending conditions [21,79,80].

### 2.6. Performance Metrics

**Figure 3:**
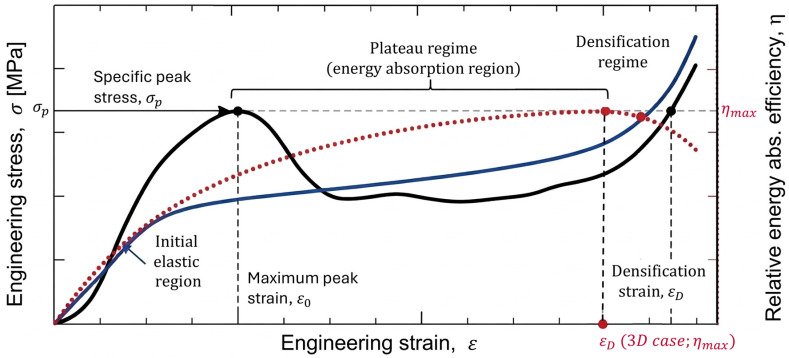
Comparison of methodologies used to determine the compressive stress–strain behavior and densification strain (*ε_D_*) in cellular structures. 2D Honeycomb (black curve) applies to 2D structures exhibiting an extended, distinct plateau; here, *ε_D_* is defined as the point where the stress reaches its initial peak value (*ε*_0_) for the second time. 3D TPMS (blue curve) applies to 3D structures that lack a clear plateau; in this instance, *ε_D_* is determined from the maximum energy absorption efficiency (red curve).

Standardized performance metrics enable quantitative comparison of energy absorption behavior across different structures, materials, and loading conditions. Figure 3 illustrates key parameters such as densification strain, maximum peak strain (ε_0_) or specific stress of maximum peak (σ_p_). The most commonly used metrics are summarized below.

Densification strain (ε_D_) corresponds to the onset of rapid stress increase due to structural compaction. For 2D structures (e.g., honeycombs), it is defined as the displacement where compressive force reaches the initial peak value. For 3D structures (e.g., TPMS), which lack a clear plateau, ε_D_ is determined from the maximum of the relative energy absorption efficiency curve, marking the transition to densification. Higher ε_D_ values indicate extended energy absorption stroke [15].

#### Specific Energy Absorption (SEA)

SEA is the most widely used metric for comparing energy absorbers and quantifies energy absorbed per unit mass [82]:

$$SEA=\frac{E_{abs}}{m}$$

where $E_{abs}$ is the total energy absorbed (area under the force–displacement curve up to ε_D_) and *m* is the specimen mass. SEA is typically reported in J/g or kJ/kg, with reported values ranging from 2 to 5 J/g for honeycombs to over 13 J/g for optimized TPMS structures [38,83].

Absorbed energy (*E_abs_*) is defined as the area under the stress–strain (σ-ε) curve up to densification [84]:

$$E_{abs}=\int_{0}^{\varepsilon_{D}}\sigma \left(\varepsilon \right)d\varepsilon$$

Peak crushing force (*F_p_*) represents the maximum load during compression and is obtained directly from the force–displacement curve. Lower *F_p_* values are desirable for protective applications, though trade-offs with total energy absorption exist. Graded structures can reduce *F_p_* while maintaining performance [85,86,87].

Average force for compression (*F_m_*) is defined as the ratio between the energy absorbed by the structure and the densification point value over which the compression is achieved [88]. Here, compression is relevant only up to the point of densification, denoted by *d_s_*, if it is calculated according to the force.

$$F_{m}=\frac{1}{d_{s}}\int_{0}^{d_{s}}F\left(x\right)dx$$

When considering the average stress ($\sigma_{m}$) that occurs after the compression, the equation takes the following form:

$$\sigma_{m}=\frac{1}{\varepsilon_{D}}\int_{0}^{\varepsilon_{D}}\sigma \left(\varepsilon \right)d\varepsilon$$

Crush load efficiency (CLE) evaluates the stability of the energy absorption plateau and is defined either as $CLE=\sigma_{m}/\sigma_{p}$ or as $CLE=F_{m}/F_{p}$. Here, $\sigma_{p}$ is the maximum stress value, specific to the maximum peak in the first portion of the compression curve. Values approaching 1 indicate ideal, constant-force energy absorption. Higher CLE values reflect improved efficiency and reduced load fluctuations [89,90].

Energy absorption efficiency ($\eta_{abs}$) compares the actual energy absorbed up to the densification point to the theoretical maximum. For curves normalized by length (plotted as $L/L_{0}$), the theoretical maximum energy is the area of a rectangle of unit length (from $L/L_{0}=0\;$ to $L/L_{0}=1$) and width $\sigma_{p}$, so the maximum energy is $\sigma_{p}\cdot 1$. Thus,

$$\eta_{abs}=\frac{E_{abs}}{\sigma_{p}\cdot 1}$$

Alternatively, when using a force–displacement curve, the theoretical maximum energy is given by the product of peak force $F_{p}\;$ and total compression displacement d up to densification. Thus,

$$\eta_{abs}=\frac{E_{abs}}{F_{p}\cdot d}$$

In both formulations, $\eta_{abs}$ equals the area under the experimental curve (absorbed energy) divided by the corresponding rectangular area (maximum possible energy) [91,92,93].

Specific stress of the linear plateau (*σ*_s_) is defined as the integral of the stress within the region between the initial peak stress and the onset of densification. This parameter reflects the stability and effectiveness of energy absorption in the plateau region [16,82]:

$$\sigma_{s}=\frac{\int_{\varepsilon_{0}}^{\varepsilon_{D}}\sigma (\varepsilon )d\varepsilon }{\varepsilon_{D}-\varepsilon_{0}}$$

Shape recovery ratio (*R_r_*) is used when dealing with flexible, reusable structures and evaluates structural recovery after deformation:

$$R_{r}=\frac{\left(h_{r}\;-\;h_{c}\right)}{(h_{0}\;-\;h_{c})\;}\times \;100\%$$

where *h*_0_ is the initial height, *h_c_* is the compressed height, and *h_r_* is the recovered height [46,94].

## 3. 2D Cellular Structures

### 3.1. Overview and Material Distribution

2D cellular structures consist of planar unit cells extruded in the third dimension, forming architectures that can be loaded either in-plane (InP) or out-of-plane (OofP). A wide range of polymeric materials has been explored, with TPU, PLA, PEEK, CF/PEEK, and ABS being the most commonly used. FDM is the predominant manufacturing technique due to its accessibility and versatility. Recent developments, including continuous fiber reinforcement and in situ heating, aim to enhance process performance [95]. Alternative methods such as SLS, PolyJet, and multi-jet fusion are employed in specialized applications requiring higher resolution, improved mechanical properties, or specific material capabilities, despite their higher cost and complexity [96,97].

### 3.2. Energy Absorption Performance Metrics

The SEA of 2D structures shows significant variability, ranging from 0.30 to 47.90 J/g, reflecting the combined influence of geometry, material, relative density, loading direction, and testing conditions. The highest values (>40 J/g) were achieved by PEEK and CF/PEEK structures under out-of-plane compression, highlighting the advantages of high-performance polymers and optimized loading configurations [98]. Volumetric energy absorption (SEA, J/cm^3^) provides complementary insight into space efficiency, with reported values ranging from 0.02 to 1.00 J/cm^3^. While mass-specific performance can be high, the low relative density of cellular structures limits volumetric absorption, highlighting a key trade-off in lightweight design for space-constrained applications [99,100].

SEA was chosen as the primary comparative metric to ensure a uniform evaluation across different relative densities and material systems, as it inherently accounts for specimen mass.

Table 2 summarizes representative 2D structures across the performance range, highlighting the relationship between architecture, material selection, and energy absorption capacity.

However, it must be noted that the wide variability in these reported SEA values also reflects the lack of standardized testing protocols mentioned in the introduction, as different strain rates and boundary conditions significantly affect the perceived energy absorption capacity.

### 3.3. Structural Topology and Deformation Mechanisms

**Figure 4:**
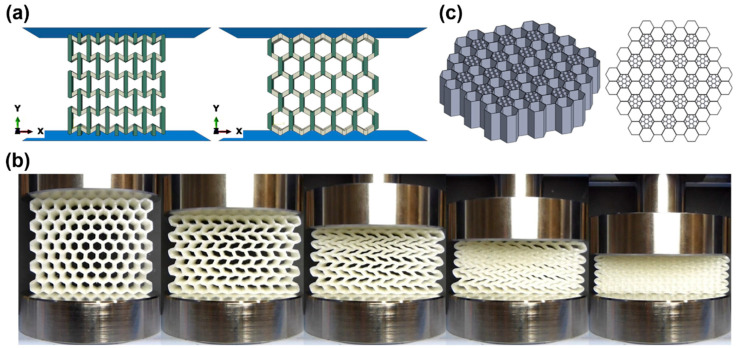
(**a**) Finite element models (FEM) of re-entrant and hexagonal cellular structures, showing reinforcement locations (green: reinforcement; gray: nylon). (**b**) Cellular collapse behavior during quasi-static compression of a medium-density uniform array. (**c**) Pomelo peel-inspired honeycomb structure: (**left**) 3D view and (**right**) top-plane view of the structural arrangement. Reproduced from ref. [108] (**a**), ref. [86] (**b**) and ref. [15] (**c**).

The dataset covers diverse topological families with distinct deformation mechanisms governing energy absorption (Figure 4). Auxetic structures, particularly re-entrant honeycombs (Figure 4a, left), display inward wall rotation and synclastic deformation, enhancing energy absorption through combined bending and stretching [96,98,108]. Hierarchical variants shift deformation toward stretching-dominated behavior and further improve performance, with multi-scale designs enabling combined buckling, densification, and controlled fracture mechanisms [96,112]. Hexagonal honeycombs (Figure 4a, right), the most common topology, exhibit elastic buckling, plastic hinge formation, and progressive folding (Figure 4b) [53,95,101]. Under in-plane compression [45,109], deformation is localized, whereas out-of-plane loading leads to more uniform collapse and higher efficiency [98,101].

**Figure 5:**
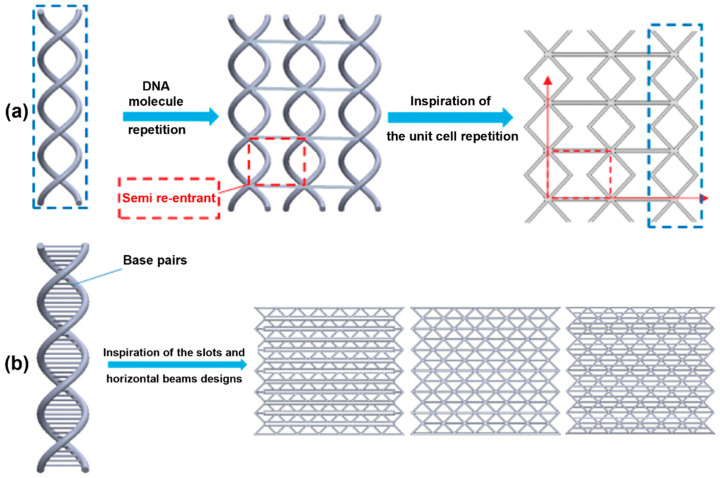
(**a**) Unit cell repetition pattern. (**b**) Schematic design of slots and horizontal beams inspired by the DNA molecular structure. Reproduced from ref. [115].

Bioinspired architectures, including bamboo-, pomelo-peel- (Figure 4c), DNA- (Figure 5), cactus-, and snake-like designs, leverage segmented and layered configurations to promote progressive failure and improved energy dissipation. For example, bamboo-inspired structures reach SEA values of 24.8 J/g [101], while double-layered helix honeycombs achieve stable layer-by-layer compression and enhanced SEA (6.4 J/g) [113].

Finally, hybrid topologies demonstrate additional gains, with windmill-like structures combining star and inverted hexagonal elements achieving 60–110% higher SEA than conventional PA12 honeycombs, highlighting the benefits of multi-geometry design strategies [97].

### 3.4. Material Selection and Performance Relationships

Material selection strongly governs both the magnitude and mechanisms of energy absorption in 2D structures. High-performance thermoplastics (PEEK, CF/PEEK) achieve the highest SEA values (41.0–47.9 J/g under OofP loading) due to superior strength and ductility; however, fiber reinforcement may induce brittleness and premature failure in some cases, reducing performance relative to unreinforced PEEK [98].

Commodity thermoplastics (PLA, ABS, PETG) offer cost-effective solutions, with PLA exhibiting SEA values from 1.08 to 24.8 J/g depending on topology, density, and loading conditions [45,101,109,110]. Its semi-brittle behavior favors single-impact applications but limits reusability. Instead, elastomers such as TPU enable recoverable and reusable energy absorption, with a reported SEA of 1.44 J/g and ~47% efficiency, along with stable shape recovery after repeated loading [28,53,86]. However, their lower stiffness reduces peak load capacity, requiring application-specific design optimization [116].

Multi-material systems combine complementary properties, with PLA/TPU hybrids achieving 2–3 times higher energy absorption than single materials due to synergistic stiffness–ductility interactions and progressive failure modes. Interfacial bonding remains critical and is often evaluated via DCB and ENF testing [105]. Composite reinforcement strategies further enhance performance. Continuous carbon fiber-reinforced PLA achieves SEA values of 5.32 J/g with added shape memory functionality [95], while short fiber composites (PA12 + CF, PLA + GF, PA + GF) show performance dependent on fiber orientation, volume fraction, and interfacial adhesion [99,104,108].

### 3.5. Loading Direction Effects

Loading direction (InP vs. OofP) has a dominant effect on energy absorption, with OofP loading typically yielding 5–10 times higher SEA for identical structures [98]. This difference arises from distinct deformation mechanisms: InP loading is governed by localized bending and buckling of cell walls, often forming weak deformation bands [96], whereas OofP loading engages the full cell wall cross-section, promoting more uniform stress distribution and higher efficiency [101].

For example, hexagonal PEEK structures exhibit SEA values of 43.6 J/g (OofP) versus 5.6 J/g (InP), with similar trends for CF/PEEK (47.9 vs. 3.4 J/g), chiral (41.0 vs. 4.8 J/g), and re-entrant (43.0 vs. 4.4 J/g) geometries [98]. These results highlight the importance of aligning structural orientation with loading conditions.

Functionally graded structures further enhance performance by tailoring deformation progression. Honeycombs with 2–5 stages of wall thickness variation show improved energy absorption compared to uniform designs, as grading promotes sequential collapse and extended plateau regions [86]. Predictive models incorporating probabilistic approaches have been developed to optimize grading strategies for specific loading scenarios [19].

### 3.6. Foam-Filling and Hybrid Strategies

Foam filling is an effective strategy for enhancing energy absorption in 2D cellular structures, though its performance is strongly material-dependent. For glass fiber-reinforced auxetic honeycombs, foam filling increased absorbed energy by 20% in PLA and 70% in PA, due to lateral support that delays buckling and promotes uniform deformation [19]. However, improvements are not universal. In three-point bending, TPU-based honeycombs showed increased specific energy absorption (SEA) with foam filling, whereas PLA structures exhibited reduced performance. This behavior reflects stiffness mismatch: when the cellular structure is significantly stiffer than the foam, the latter adds mass without effective reinforcement, lowering SEA [21].

Hybrid approaches combining FDM with supercritical CO_2_ (scCO_2_) foaming have emerged as promising alternatives. TPU honeycombs fabricated via this method achieved SEA values of 1.04 J/g, compared to 0.83 J/g for unfoamed structures (~25% improvement), due to hierarchical porosity enabling multi-scale energy dissipation [55]. The scCO_2_ process introduces closed-cell porosity within filaments, reducing density while maintaining structural integrity and improving integration compared to conventional foam filling. However, careful process control is required, as excessive foaming may degrade mechanical properties [55].

## 4. 2D Sandwich Structures

### 4.1. Overview and Configuration Characteristics

2D sandwich structures combine the simplicity and cost-effectiveness of planar cellular cores with the structural efficiency of sandwich configurations, consisting of honeycomb, auxetic, or rectangular cores bonded between face sheets [21,55,56,76,115,117,118]. These architectures provide high bending stiffness and enhanced energy absorption. Common material systems include TPU, TPU + PU foam, PP + CFRP, PETG + CF, PETG, and PLA + CF [56,76,115,118]. FDM is the predominant fabrication method due to its accessibility, although hybrid approaches such as FDM combined with scCO_2_ foaming have also been investigated [119].

### 4.2. Energy Absorption Performance

The SEA of 2D sandwich structures shows substantial variability, reflecting the influence of core topology and material combinations. Notably, accordion-type cellular cores achieved the highest SEA values under quasi-static compression within the dataset [118]. Available volumetric SEA data are limited but comparable to bulk 2D structures, indicating that face sheets primarily enhance bending stiffness and structural integrity rather than volumetric energy absorption [55]. Table 3 summarizes the most representative 2D sandwich structures, highlighting variations in core architecture and material systems.

### 4.3. The Effects of Core Topology and Material Selection

**Figure 6:**
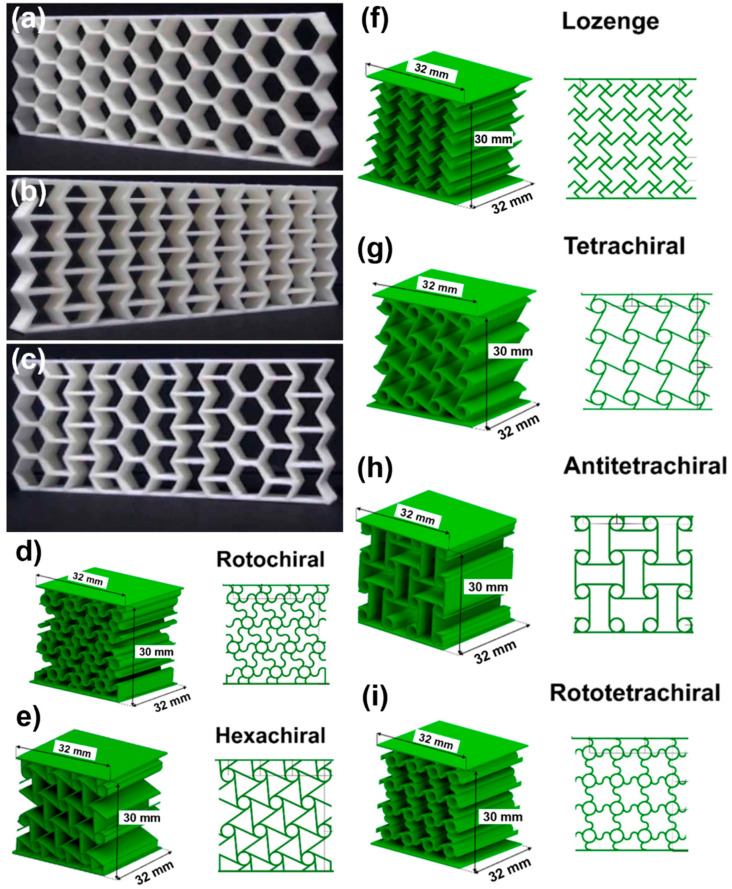
(**a**–**c**) 3D-printed mechanical metamaterials used for compression testing: (**a**) hexagonal structure, (**b**) re-entrant auxetic structure, and (**c**) AuxHex structure. (**d**–**i**) 3D CAD models and corresponding front views of rotochiral (**d**), hexachiral (**e**), lozenge (**f**), tetrachiral (**g**), antitetrachiral (**h**), and rototetrachiral (**i**) geometries. Reproduced from ref. [121] (**a**–**c**), and ref. [122] (**d**–**i**).

Core topology governs deformation and energy absorption in 2D sandwich structures, with hexagonal (Figure 6a), auxetic (Figure 6b), and rectangular cores showing comparable absorption but distinct mechanisms: hexagonal cores exhibit localized buckling, whereas auxetic geometries promote synclastic deformation and improved damage tolerance [56]. Hybrid auxetic and other complex designs (Figure 6c–i) further enhance indentation resistance through more uniform load distribution, with optimal performance at moderate relative densities (20–40%) [21,56,121,122]. Foam filling produces material-dependent effects, increasing energy absorption in TPU-based structures but reducing performance in PLA due to stiffness mismatch, while in other geometries, gains of up to 50% in absorbed energy have been reported [56,120].

**Figure 7:**
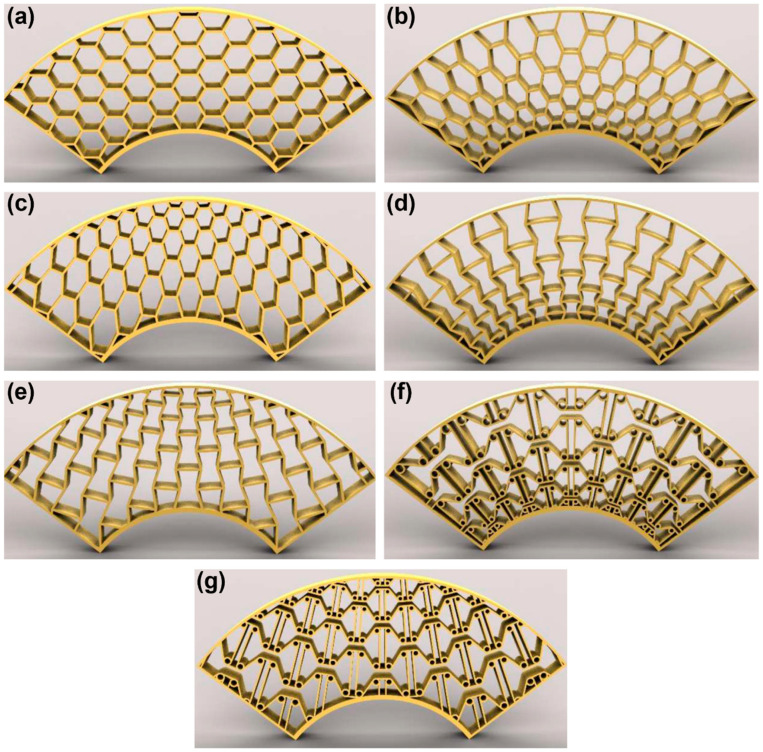
3D CAD models of the lattice structures investigated in this study: (**a**) uniform honeycomb, (**b**) functionally graded (FG) honeycomb I, (**c**) FG honeycomb II, (**d**) FG re-entrant I, (**e**) FG re-entrant II, (**f**) FG RCA I, and (**g**) FG RCA II. Reproduced from ref. [123].

Material selection further influences performance by balancing stiffness and recoverability. Elastomeric TPU cores enable high energy absorption and shape recovery for reusable applications, though their low stiffness reduces bending rigidity compared to rigid thermoplastics [55]. Carbon fiber reinforcement enhances stiffness and strength, as demonstrated in PETG + CF sandwich structures, while continuous fiber reinforcement provides superior mechanical performance compared to short fibers, albeit with increased manufacturing complexity and strong dependence on fiber orientation relative to the loading direction [56,95]. Loading direction and impact energy also play critical roles: out-of-plane loading induces face sheet bending and core compression, with auxetic cores showing improved damage tolerance [117], while low-velocity impacts produce largely elastic responses, and higher energies activate combined face sheet deformation, core crushing, and fiber fracture mechanisms [56,76]. The effect of the compression direction becomes evident in sandwich structures with core designed for integration onto circular surfaces [123]. In such cases (Figure 7), the structure must replicate the curvature of the outer walls to ensure efficient energy absorption under multidirectional impact loading.

## 5. 3D Lattice Structures

### 5.1. Overview, Structural Diversity and Energy Absorption Metrics

3D lattices are spatial cellular architectures in which load-bearing geometry extends in all directions, enabling distributed deformation, improved load transfer, and more isotropic mechanical responses compared to planar systems [124]. These structures are broadly classified into strut-based lattices, composed of interconnected beam elements (e.g., BCC, FCC, octet, Kelvin) [41,81,125], and sheet- or surface-based architectures, including TPMS geometries characterized by continuous surfaces and complex connectivity [41,81,125,126].

Material selection in 3D lattices is more diverse than in 2D systems, with PLA being the most common, followed by TPU, PA, ABS, and multi-material systems such as VeroWhitePlus + Agilus [127]. This diversity reflects the broader range of AM technologies employed, including FDM, SLA, DLP, PolyJet, and MJF [41,52,61,128,129]. In particular, vat photopolymerization techniques (SLA, DLP) are widely used due to their ability to accurately reproduce complex geometries, such as TPMS, with high resolution and surface quality [130].

The SEA of 3D lattices spans a wide range (up to 38.90 J/g under dynamic loading), generally lower than peak values reported for 2D structures [69,72]. However, this comparison must consider their typically lower relative densities and more isotropic behavior. In contrast, volumetric energy absorption (SEA, J/cm^3^) is more extensively reported, with values up to ~5.76 J/cm^3^, highlighting the superior space efficiency of 3D lattices at higher densities [128,131].

**Figure 8:**
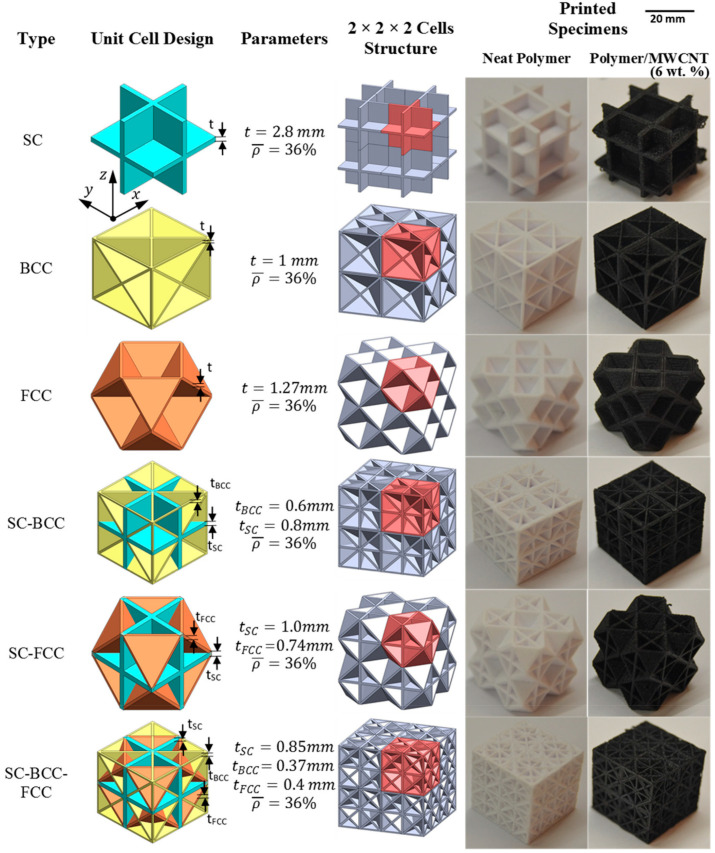
The unit cell design, cell design parameters, CAD models and printed specimens of various elementary and hybrid plate-lattice structures. Note that all plate lattices have the same relative density of 36%. Reproduced from ref. [81].

Energy absorption efficiency ranges from 27% to 93.6%, with the highest values observed in TPU-based double arrowhead plate-lattice (ST-DAPL) structures fabricated via SLS. Such high efficiencies indicate stable crushing behavior with minimal peak force transmission, which is critical for protective applications [62,132]. Examples of various plate-lattice structures, along with their unit cell design, cell design parameters, and CAD models, are further shown in Figure 8. Table 4 summarizes representative 3D lattice architectures, illustrating the relationships between topology, material selection, and energy absorption performance.

### 5.2. Strut- and Sheet-Based Lattice Structures

**Figure 9:**
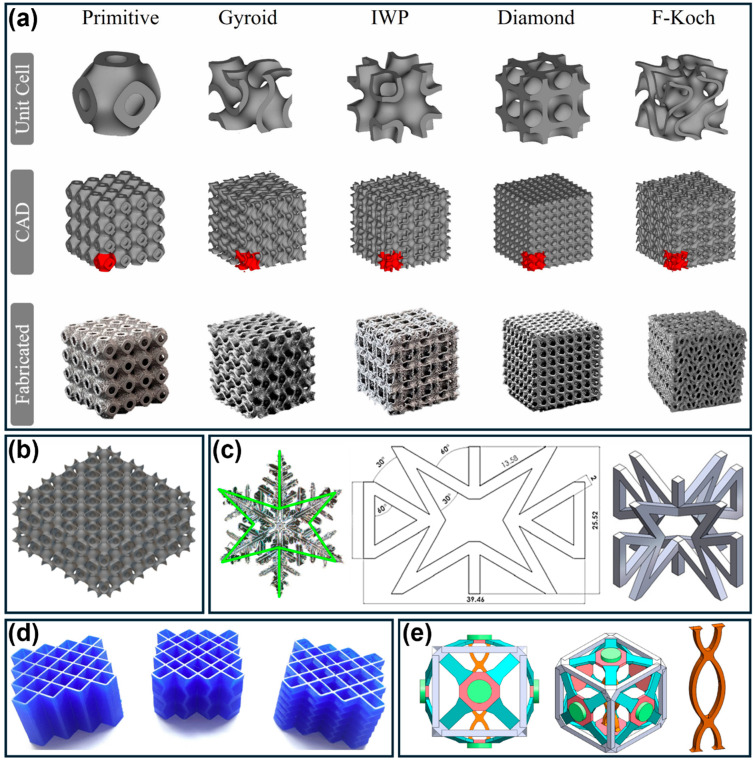
(**a**) Architectural designs showing unit cells, CAD lattice structures, and fabricated samples of primitive, gyroid, IWP, diamond, and Fisher–Koch lattice structures. (**b**) 3D TPMS lattice arrangement for the FRD222 base cell. (**c**) Bio-inspired unit cell of a novel 3D star-triangular auxetic honeycomb (3DSTH) (all dimensions in millimeters). (**d**) FFF-printed origami honeycombs with specimens having *t* = 1.0 mm, *w* = 12.5 mm, *h* = 30.0 mm, and *e* = 1.0 (**left**), *e* = 0.7, *n* = 6 (**center**), and *e* = 0.7, *n* = 12 (**right**). (**e**) Unit cell UC-5: (**left**) side view, (**center**) isotropic view, and (**right**) DNA member. Reproduced from ref. [126] (**a**), ref. [15] (**b**), ref. [134] (**c**), ref. [133] (**d**), and ref. [131] (**e**).

**Figure 10:**
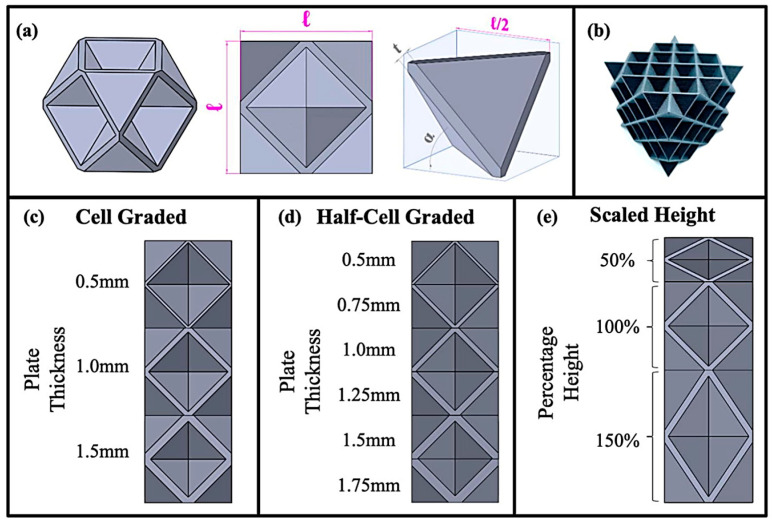
Orientation and geometry of: (**a**) a single octet unit cell (isometric, front, and sectional views), (**b**) isometric view of the 3D-printed baseline lattice, (**c**) cell-graded design, (**d**) half-cell-graded design, and (**e**) scaled-height design. Reproduced from ref. [52].

Strut- and sheet-based lattices (Figure 9 and Figure 10a,b), composed of discrete beam elements connected at nodes, represent a fundamental class of 3D energy absorbers. Among these, the octet-truss topology, based on a face-centered cubic arrangement with diagonal struts, has been widely studied due to its high specific stiffness and strength [52,84,91,125,128,151]. Octet structures fabricated from PA via FDM achieved SEA values of 13.8 J/g, with performance governed by strut geometry and relative density [52]. Their deformation behavior is controlled by nodal connectivity (Z). Lattices with Z < 12 are bending-dominated, exhibiting lower stiffness but enhanced energy absorption through progressive buckling, whereas Z ≥ 12 structures are stretching-dominated, offering higher stiffness but potentially more brittle failure [125]. The octet-truss (Z = 12) represents a transitional configuration balancing stiffness and energy absorption [84].

Material and architectural modifications further enhance performance. Graphene-reinforced PMMA octet lattices (≤0.10 wt%) fabricated via SLA achieved SEA values up to 38.9 J/g, the highest reported for 3D structures, along with improved modulus and failure strength, outperforming conventional lightweight materials on a mass basis [69]. Hybrid PA/PA-CF lattices exhibited comparable SEA (~13.7 J/g) to pure PA while offering improved stiffness, highlighting the potential of tailored material distribution [52].

Finally, geometric parameters also have a strong influence on performance, with energy absorption varying from 0.02 to 1.80 MJ/m^3^ depending on strut length, radius, and density—a variation of nearly two orders of magnitude [16]. This sensitivity underscores the importance of geometry optimization for application-specific design.

### 5.3. Triply Periodic Minimal Surface Structures (TPMS)

TPMS structures constitute a promising class of 3D energy absorbers, defined by smooth, continuous surfaces with zero mean curvature that partition space into two interpenetrating domains. Common topologies (Figure 9a) include gyroid, primitive (Schwarz P), diamond, and IWP geometries [15,87,129,136,146,147]. Among these, the gyroid has received particular attention due to its favorable combination of mechanical performance, manufacturability, and energy absorption. Its continuous geometry promotes uniform stress distribution, progressive deformation, and extended plateau regions [3,23]. For example, PA-CF gyroid lattices (10 mm cell size, 2 mm wall thickness) achieved SEA values of 13.06 J/g (±0.15), outperforming both 3D truss and conventional 2D lattices [38]. Furthermore, studies on FRD TPMS lattices (Figure 9b) fabricated via FDM show that smaller cell sizes enhance deformation uniformity and energy absorption efficiency, although at increased manufacturing cost [15]. Advanced multi-material designs, such as interpenetrating phase composites combining rigid (VeroWhitePlus—Stratasys, Eden Prairie, MN, USA) and soft (Agilus 30—Stratasys, Eden Prairie, MN, USA) networks, achieved volumetric SEA values of 1.75 J/cm^3^ and retained more than 45% of stiffness and strength after cyclic loading. Enhanced performance arises from interfacial mechanisms including crack deflection and ligament bridging [68].

Bioinspired TPMS gyroid composites further improve energy absorption, exhibiting extended plateau regions and bending-dominated deformation. Gibson–Ashby analysis indicates that size effects become significant at relative densities below 15%, providing guidance for optimized design [136,138,147].

### 5.4. Auxetic 3D Lattice and Origami-Inspired Structures

3D auxetic lattices, characterized by negative Poisson’s ratios, exhibit unique energy absorption through lateral expansion during compression. A 3D star-triangular auxetic honeycomb (Figure 9c), fabricated from TPU and PA12 via MJF, showed strong material dependence, with SEA values of 0.0543 J/g (TPU) and 0.616 J/g (PA12), highlighting the influence of stiffness and failure mode [134]. Auxetic behavior increases contact area during compression, promoting energy dissipation via friction and progressive densification. While this mechanism enhances dissipation, it is highly sensitive to manufacturing quality. Consequently, the overall effectiveness of auxetic designs remains debated, with performance strongly dependent on relative density, loading rate, and material properties [62,135,152].

Origami-inspired 3D structures exploit folding principles to create deployable energy absorbers with tunable mechanical responses. Square origami honeycombs (Figure 9d), fabricated from TPU via FDM, achieved 49% energy absorption efficiency, with folded configurations enabling smoother stress–strain behavior and improved deformation control. The use of elastomeric materials allows shape recovery after compression, supporting repeated use. These architectures enable programmable deformation sequences and compact storage; however, their geometric complexity poses manufacturing challenges, particularly in maintaining precise fold angles and avoiding defects at crease regions [133].

Finally, re-entrant 3D honeycombs, a common auxetic configuration, offer controlled deformation through cell wall rotation and densification, though reported SEA values are limited [62,134]. Other complex architectures, such as NSS structures (Figure 9e), show potential for extended service life despite modest energy absorption performance [131].

### 5.5. Graded and Functionally Designed 3D Lattices

Functionally graded 3D lattices (Figure 10c–e), characterized by spatial variations in topology, density, or material composition, represent an advanced strategy for optimizing energy absorption [52,63,85,87,153]. Hybrid graded foams combining variations in binder size and shell thickness achieved volumetric SEA values of 2.18 J/cm^3^, corresponding to improvements of 125%, 185%, and 34% over uniform, graded binder, and graded thickness foams, respectively [63]. This enhanced performance is attributed to optimized stress distribution and sequential failure mechanisms. Medium binder sizes promote uniform stress transfer and simultaneous failure of binder and shell, maximizing energy absorption, whereas excessively small or large binders lead to localized stress concentrations and reduced efficiency [63]. Graded TPMS structures, incorporating spatial variations in wall thickness or cell size, were shown to achieve volumetric SEA values of 1.655 J/cm^3^. Although lower than hybrid graded foams, they offer advantages in terms of smooth geometry, reduced stress concentrations, and improved manufacturability [63].

### 5.6. Comparative Performance of 3D vs. 2D Structures

Direct comparison of 2D and 3D energy-absorbing structures highlights complementary advantages and inherent trade-offs. Although 2D architectures achieve higher peak SEA values (up to 47.9 J/g) compared to 3D lattices (up to 38.9 J/g), this difference largely reflects dissimilar loading conditions—out-of-plane compression for 2D versus dynamic uniaxial loading for 3D systems [69,98]. Under comparable conditions and relative densities, 3D lattices generally exhibit more isotropic behavior and predictable deformation due to their fully distributed load-bearing networks [52,98].

A more balanced comparison is provided by volumetric energy absorption, where 3D architectures consistently outperform 2D structures [62,100,103,128]. This advantage arises from the efficient distribution of material throughout the volume, enabling improved load transfer and enhanced space efficiency.

From a design perspective, 2D structures remain advantageous for applications with well-defined loading directions, offering simplicity, lower manufacturing cost, and high directional performance [101,110]. In contrast, 3D lattices are preferred for complex or multi-directional loading scenarios, where isotropic properties, higher volumetric efficiency, and tunable deformation mechanisms are required [84,128].

## 6. 3D Sandwich Structures

### 6.1. Overview and Architectural Characteristics

3D sandwich structures combine conventional sandwich panel design with volumetric cellular cores, including TPMS, strut-based lattices, and other complex architectures. Compared to 2D sandwich systems, these structures provide enhanced out-of-plane performance while maintaining high bending stiffness and low weight. Under flexural loading, three primary failure modes are observed: face yield, core shear, and indentation. Face yield occurs when face sheet stresses exceed material limits, typically in thin-faced or stiff-core configurations; core shear involves shear band formation and may lead to catastrophic failure; and indentation results from localized core crushing under concentrated loads. These mechanisms directly influence energy absorption, with face yield providing limited dissipation, core shear leading to abrupt failure, and indentation offering moderate absorption through localized deformation [73,79,80,85,119].

Material combinations are more limited than in bulk 3D structures, including UTR8119 resin with aluminum face sheets [85], PA with glass fiber reinforcement (PA + GFR) [73], TPU/PVDF blends [119], and conventional polymers such as ABS [79] and PLA [80]. Fabrication methods include FDM [79,80], SLA [85], SLS [73] and hybrid FDM with scCO_2_ foaming [119], reflecting diverse approaches to producing sandwich structures with complex cores.

### 6.2. Energy Absorption and Bending Performance

SEA data for 3D sandwich structures, though limited, falls within a relatively narrow range of 2.20–4.92 J/g, indicating consistent and predictable performance governed primarily by core topology and face sheet properties [85,119]. Volumetric SEA remains low (e.g., 0.034 J/cm^3^ for NSMS PA + GFR core structures), reflecting the high porosity of sandwich cores and their optimization for flexural performance rather than volumetric efficiency [73].

Bending stiffness ranges from 1698.5 to 2950 N/mm, highlighting the high flexural rigidity achievable with 3D sandwich architectures. The maximum value (2950 N/mm) was reported for cubic PLA cores fabricated via FDM [80], while TPMS cores (Primitive, Neovius, IWP) exhibited values between 1698 and 1914 N/mm, with Neovius showing the highest stiffness [79]. Table 5 summarizes representative 3D sandwich structures, illustrating the relationships between core topology, material selection, and mechanical performance.

### 6.3. TPMS Cores in Sandwich Structures

TPMS topologies have proven effective as core architectures in 3D sandwich structures, owing to their smooth geometry, uniform stress distribution, and high bending resistance. Functionally graded lattice beams (FGLBs) with TPMS cores show that gradient design significantly influences bending response, energy absorption, and failure modes [85].

Comparative studies with aluminum face sheets and FRP reinforcement indicate strong topology dependence. Gyroid cores achieved the highest SEA (4.92 J/g), followed by FGLBs and primitive structures, attributed to their higher surface area and more uniform stress distribution [85,146]. Gradient designs further enhance performance by increasing SEA without significantly raising peak load force (PLF), while flexural behavior remains governed by face yield, core shear, and indentation mechanisms.

FRP reinforcement provides substantial improvements, with SEA increasing by up to 31.32% (from over 2844 to almost 3735 J/kg) in graded metamaterial beams without significant weight penalty [85]. For thin-faced sandwich structures, Neovius cores offer superior bending strength and energy absorption compared to primitive and IWP geometries, although bending stiffness is more strongly influenced by geometric parameters and relative density than topology [79].

### 6.4. Strut-Based Cores in Sandwich Structures and Novel Core Topologies

Strut-based lattice cores, including cubic, octet, and Isomax topologies (Figure 9), provide alternative architectures for 3D sandwich structures with distinct mechanical responses. Comparative bending and low-velocity impact tests demonstrate that both topology and geometric parameters strongly influence failure mechanisms and energy absorption [80]. Performance is highly dependent on structural configuration. For beams with a length-to-span ratio (Ls) = 3, octet cores (30% relative density) exhibit higher quasi-static energy absorption, albeit with increased contact loads. At Ls = 1.8, cubic and Isomax cores (50% relative density) show superior performance, highlighting the importance of matching topology to loading conditions and geometry [80]. Under high-energy impact, octet cores provide the highest energy absorption, while build orientation significantly affects multi-impact resistance and damage tolerance [80].

Novel core designs further expand performance potential. Double curved-beam (NSMS) cores, fabricated from PA + GFR via SLS, exhibit layer-by-layer failure under repeated loading, creating a damage isolation effect that enhances impact resistance. This progressive failure mechanism contrasts with conventional shear-dominated collapse in honeycombs and is particularly advantageous for multi-impact applications [73].

### 6.5. Hierarchical and Hybrid Core Strategies

Hierarchical cellular structures, combining macro-scale honeycomb geometry with micro-scale foam porosity, represent an effective strategy for enhancing sandwich performance. TPU/PVDF honeycomb cores fabricated via FDM with scCO_2_ foaming achieved SEA values of 2.20 J/g, with energy absorption increases of 152.75%, 67.33%, and 56.91% across three compression directions compared to unfoamed TPU structures [119]. Furthermore, gradient stiffness designs, obtained by varying TPU/PVDF composition, exhibited distinct yield patterns relative to homogeneous structures. A six gradient configuration produced multiple energy absorption plateaus, enabling sequential energy dissipation. These results highlight the potential of combining hierarchical and graded design with advanced manufacturing to optimize structural performance [119].

## 7. Emerging 4D-Printed Structures

### 7.1. Concept and Enabling Technologies

4D printing represents a transformative extension of additive manufacturing, introducing time-dependent functionality to energy-absorbing structures [37,154]. While many 2D and 3D architectures exhibit reusability, they are often not explicitly classified as 4D systems nor systematically studied in this context. Unlike conventional static structures, 4D-printed systems can autonomously change shape or properties in response to external stimuli (e.g., temperature, moisture, magnetic fields), addressing the key limitation of single-use energy absorbers [46,122,155,156]. This capability is enabled by integrating shape memory polymers and alloys with advanced manufacturing techniques [157,158,159]. These materials exhibit a shape memory effect (SME), allowing structures to undergo large deformations during impact and subsequently recover their original geometry upon activation (e.g., thermal stimulus above the glass transition temperature, *T_g_*), restoring functionality for repeated use [121,123,160]. Recent studies report shape recovery ratios exceeding 90–99% after severe deformation (50–80% strain), highlighting the potential of 4D-printed systems for intelligent, adaptive, and reusable energy absorbers in automotive, aerospace, protective, and biomedical applications [161,162,163,164].

### 7.2. Shape Memory Materials and Stimuli-Responsive Mechanisms

**Figure 11:**
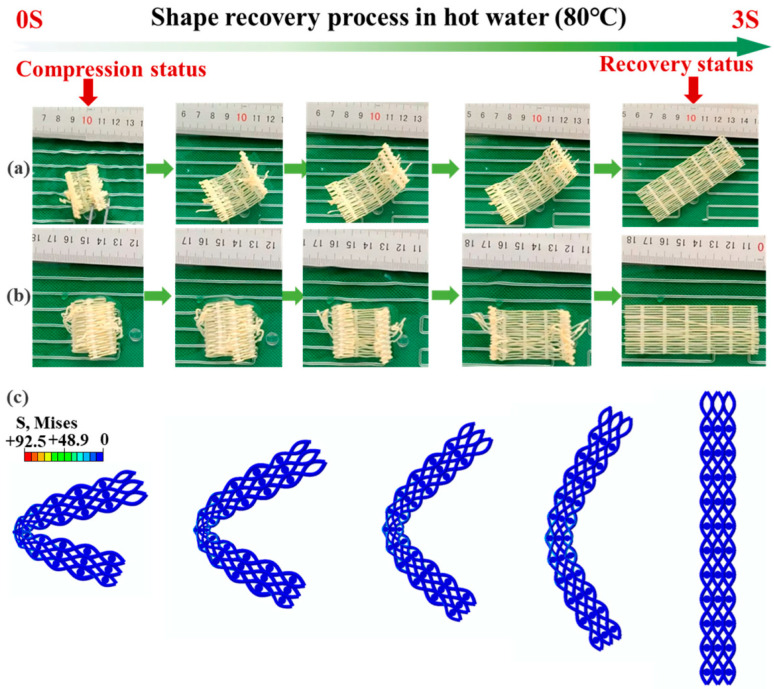
(**a**–**c**) Shape recovery behavior of a braided mechanical metamaterial: (**a**) shape recovery process of the 4D-printed braided structure after folding, (**b**) shape recovery process after curling, and (**c**) finite element simulation results of shape recovery after folding. Reproduced from ref. [163].

Shape memory polymers (SMPs) constitute the primary material platform for 4D-printed energy absorbers, with PLA and TPU being the most widely investigated systems. These materials exhibit *T_g_* temperatures typically between 55 and 70 °C, enabling activation via thermal stimuli such as hot water, convective heating, or infrared radiation. Their shape memory behavior arises from the transition between a rigid glassy state (below *T_g_*) and a compliant rubbery state (above *T_g_*), where increased molecular mobility facilitates recovery [37,121,123]. The shape memory cycle involves programming at elevated temperature, fixation upon cooling below *T_g_*, and recovery upon reheating, driven by entropic forces, as shown in Figure 11. Both hot and cold programming approaches have been developed, with cold programming offering practical advantages for real-world applications (e.g., automotive systems) while maintaining recovery ratios above 90% and preserving mechanical integrity [15,37].

Shape memory hybrid systems, combining SMP matrices with continuous fiber reinforcement, represent an emerging direction for enhancing mechanical performance and durability. Continuous carbon fiber-reinforced PLA/TPU lattices have demonstrated shape recovery ratios of over 99% in the first cycle and more than 90% after repeated loading, with fibers improving stiffness and mitigating failure [165]. Similarly, continuous fiber-reinforced honeycomb structures achieved up to 95% recovery at 50% strain, with rapid recovery (~20 s) upon thermal activation [75]. Beyond fiber reinforcement, nanoparticle incorporation enables multifunctionality; for example, nano-Fe_3_O_4_ SMP composites allow magnetic-responsive actuation in addition to thermal triggering, expanding the operational capabilities of 4D-printed energy-absorbing systems [166].

### 7.3. Geometric Design Strategies for 4D Energy Absorbers

Auxetic metamaterials represent one of the most extensively studied platforms for 4D energy absorption due to their negative Poisson’s ratio and unique deformation mechanisms. Re-entrant geometries enable lateral contraction during compression, enhancing energy dissipation through synclastic curvature and densification [121]. Dual-material auxetic meta-sandwiches combining soft materials and SMPs demonstrated improved performance, with optimized configurations achieving 14% higher energy absorption by reducing stress concentration [37]. Comparative studies further show that auxetic structures outperform conventional honeycombs in force capacity, energy dissipation, and shape recovery (up to 100%), owing to buckling-dominated deformation that minimizes structural damage [121].

**Figure 12:**
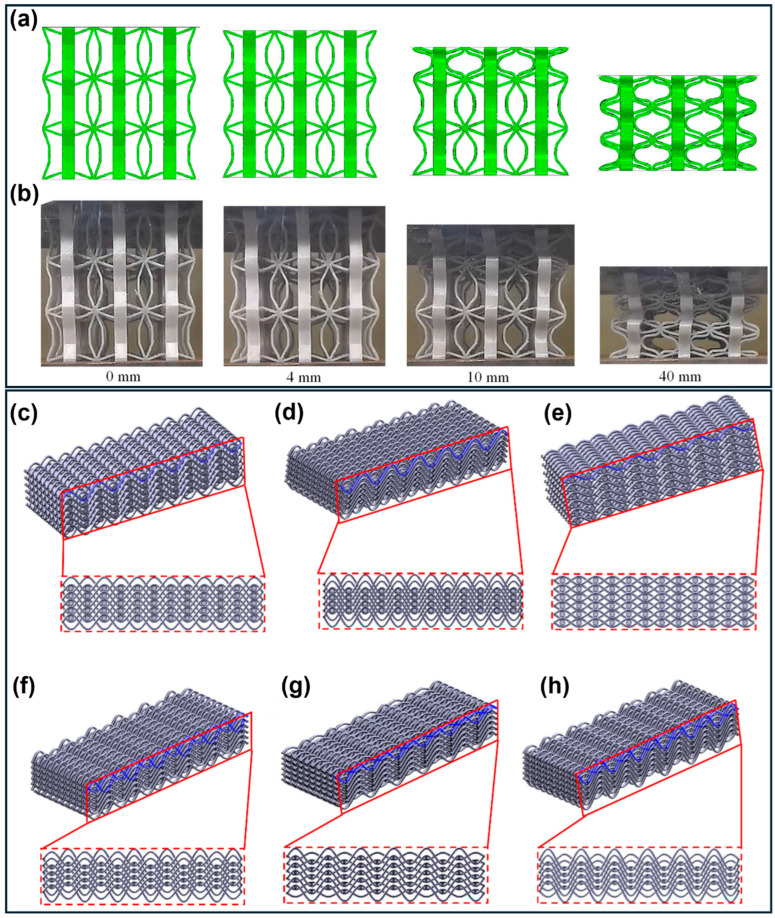
(**a**,**b**) Deformation patterns of the parent model “A” obtained from finite element analysis (FEA) (**a**) and experiment (**b**). (**c**–**h**) Braided structures with different design parameters: (**c**) Type 1, (**d**) Type 2, (**e**) Type 3, (**f**) Type 4, (**g**) Type 5, and (**h**) Type 6. Reproduced from ref. [160] (**a**,**b**), and ref. [163] (**c**–**h**).

Zero Poisson’s ratio (ZPR) metamaterials (Figure 12a,b) provide controlled deformation without lateral strain, offering advantages for stability and vibration isolation. Piecemeal energy absorption (PEA) strategies enable staged deformation with tunable peak forces, achieving energy absorption up to four times higher than conventional auxetic designs [160]. Comparative analyses of negative (NPR), zero (ZPR), and positive (PPR) Poisson’s ratio cellular metamaterials fabricated from shape memory PLA indicate that, while NPR structures exhibit the highest recovery ratios (due to their buckling-dominated deformation, which minimizes damage to cellular elements), ZPR architectures provide superior vibration isolation across deformation stages, with all systems maintaining high shape fixation (~100%) and recovery (≈86–93%) [94].

Bio-inspired and origami-derived geometries exploit natural design principles and folding mechanisms to enhance performance. Horseshoe-inspired structures demonstrate scalable energy absorption, with increases in cell density significantly improving both total and specific energy absorption [157]. Furthermore, multi-stiffness wavy metamaterials further enable adaptive response through shape recovery features [161], origami-based designs integrate deployability and controlled buckling, enabling compact storage, enhanced energy absorption, and even multifunctionality, such as strain sensing and magnetic actuation in nano-reinforced systems [164,166].

Chiral and woven architectures introduce alternative deformation mechanisms, including compression–twist coupling and interlaced load paths. Chiral lattices (Figure 6d–i) exhibit density-dependent performance, with energy absorption increasing significantly at higher relative densities (hexachiral and rotochiral structures), while configurations such as lozenge structures achieve rapid and complete shape recovery [122]. Woven metamaterials (Figure 12c–h), inspired by textile structures, enhance toughness and enable anisotropic energy absorption, offering potential for applications such as vibration mitigation in aerospace systems [163].

TPMS-based 4D structures combine smooth geometries with shape memory functionality, enabling high energy absorption and reusability. Hybrid TPMS lattices composed of gyroid and diamond substructures demonstrated SEA values up to 29 J/g, along with near-complete shape fixation and recovery and no significant structural degradation after repeated cycles [167]. These results highlight the potential of TPMS architectures for durable, reusable energy-absorbing systems.

### 7.4. Energy Absorption Performance and Reusability

The performance of 4D-printed energy absorbers is characterized by metrics such as SEA, energy dissipation, shape fixity and recovery ratios, and cyclic stability. Recent studies indicate that 4D structures achieve comparable first-cycle performance to conventional 3D systems while offering the key advantage of reusability [37,121,160]. Bodaghi et al. [37] quantified energy partitioning in dual-material auxetic meta-sandwiches, showing that SMP lattices dissipated 1.95 J, with 0.3 J absorbed, while FlexPro lattices exhibited lower dissipation (approximately 0.088 J) but higher absorption (0.13 J). The hybrid FlexPro-SMP configuration optimized this balance (0.2795 J dissipation, 0.168 J absorption) and enabled full recovery through thermal activation. Zeng et al. [75] further demonstrated that continuous fiber-reinforced honeycomb structures achieved recovery ratios of up to 95% at 50% strain, with full recovery within 20 s and optimal performance at l = 24 mm and θ = 63°. Performance metrics of the most representative systems are summarized in Table 6.

Cyclic performance is critical for practical deployment. Dong et al. [46] reported stable recovery ratios above 90% (slightly over 99% initially) over six compression–recovery cycles in fiber-reinforced auxetic structures, with stress–strain responses stabilizing after the initial cycles. Similarly, Gisario et al. [122] showed that chiral meta-sandwiches maintain structural integrity under repeated loading, with the lozenge topology exhibiting rapid and progressively improved recovery (up to 3.5 mm by the fourth cycle). Comparable reusability has also been observed in systems not explicitly classified as 4D, particularly those based on elastomeric or shape memory materials [15,28,53,86].

Failure mechanisms strongly influence recoverability and durability. Zeng et al. [75] identified direction-dependent modes in fiber-reinforced honeycombs, with out-of-plane loading inducing progressive folding and in-plane loading producing localized shear bands; notably, structures sustained up to 50% deformation without fracture due to matrix ductility and fiber bridging. Hamzehei et al. [160] observed reduced fracture and improved cyclic consistency in scaled-down ZPR structures, indicating size-dependent damage accumulation. Xu et al. [94] further showed that NPR architectures favor buckling-dominated deformation, minimizing local damage and enabling superior recovery compared to PPR systems. These findings highlight that promoting bending and buckling over tensile failure is essential for maximizing reusability in 4D energy absorbers.

### 7.5. Computational Modeling and Design Optimization

Accurate computational modeling of 4D energy absorbers requires capturing coupled thermomechanical behavior, large deformations, and history-dependent responses of shape memory materials. Bodaghi et al. [37] developed ABAQUS/Standard finite element models combining hyperelastic constitutive laws for soft FlexPro components with elasto-plastic models incorporating cold programming for SMPs. The simulations showed close agreement with experiments, accurately predicting yield stress, plastic deformation, unloading behavior, and hysteresis in force–displacement responses. Namvar et al. [121] extended this approach to re-entrant auxetic, hexagonal, and hybrid AuxHex structures, successfully reproducing mechanical hysteresis, energy dissipation, and full recovery of residual strain under thermal activation. These validated models enable efficient parametric optimization of geometry and material properties, reducing reliance on experimental prototyping. Similarly, Serjouei et al. [157] developed coupled material–structural models for horseshoe-shaped sandwich structures, accurately predicting post-unloading deformation and guiding optimization of processing parameters (e.g., wall thickness, layer height, nozzle temperature) to maximize compressive strength and energy absorption.

### 7.6. 4D-Printed Structures as the Future of Energy Absorption Technologies

A defining advantage of 4D-printed energy absorbers is their reversibility, i.e., the ability to recover original geometry and functionality after impact via simple thermal activation, thereby challenging the conventional single-use paradigm [154]. This capability enables repeated use of components such as automotive crash structures, significantly reducing material waste and lifecycle costs [121,160]. Beyond reusability, sustainability is further enhanced through material innovation, including bio-based SMPs. For example, biomass-derived shape memory polyester lattices demonstrate effective recovery and energy absorption while offering renewable alternatives to petroleum-based systems [167]. Importantly, deformation and energy dissipation processes have been shown to be fully reversible without performance degradation, enabling durable, reusable protective systems [37].

4D-printed systems enable adaptive behavior through programmable deformation and integrated sensing. Origami-inspired shape memory metamaterials with embedded sensing capabilities can simultaneously absorb energy and monitor strain, enabling intelligent systems capable of damage detection and autonomous recovery [164]. Additionally, programmable architectures, such as piecemeal energy absorption designs, allow staged deformation with controllable peak forces through sequential unit cell densification, enabling tailored force–displacement responses for application-specific requirements (e.g., progressive crash mitigation) [160]. These advances position 4D structures as a foundation for smart, responsive energy-absorbing systems integrated with sensing and control technologies.

4D-printed architectures facilitate the integration of energy absorption with additional functionalities, including vibration damping, structural load-bearing, and electromagnetic shielding. Woven metamaterials developed for aerospace applications demonstrate combined vibration mitigation and impact absorption, addressing dual functional requirements in systems such as aircraft landing gear [163]. Similarly, fiber-reinforced shape memory composites achieve high stiffness (e.g., storage modulus of 5660 MPa) while maintaining recovery ratios above 90%, enabling structural–protective multifunctionality within a single component [165]. The incorporation of topology optimization further enhances this paradigm, enabling the automated design of structures that simultaneously satisfy constraints on energy absorption, weight, recoverability, and manufacturability.

**Figure 13:**
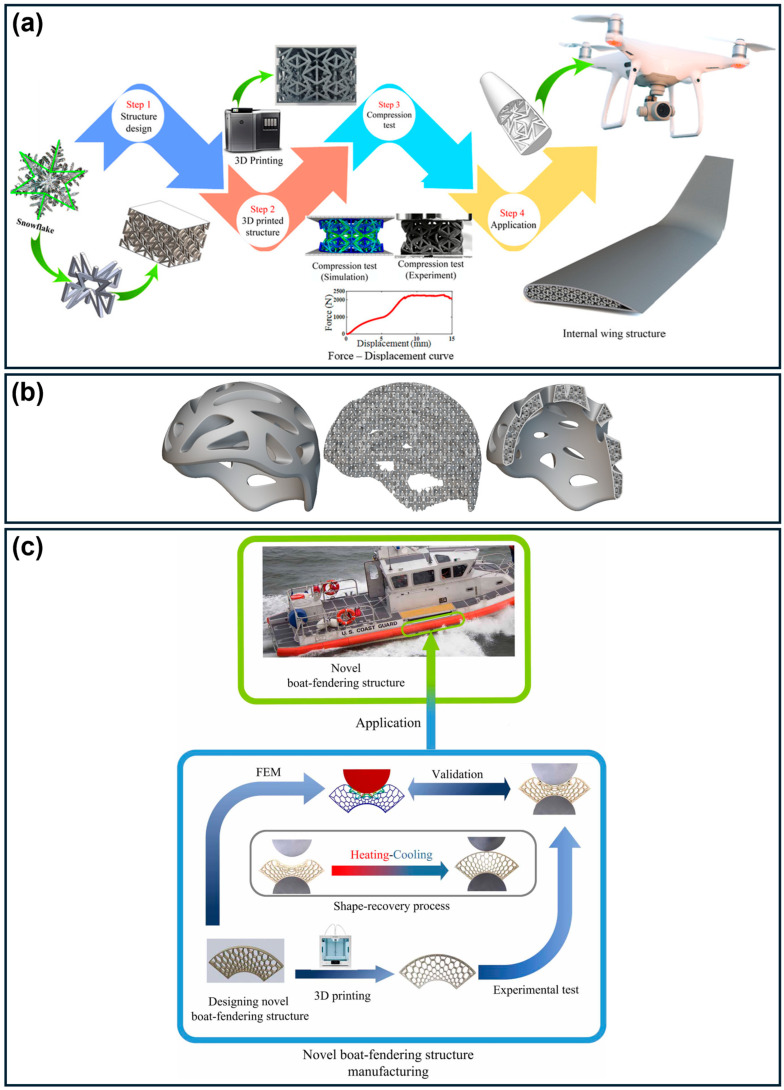
(**a**) Workflow illustrating the development of a novel 3D auxetic structure, from design to application. (**b**) Finite element model of a safety bicycle helmet: outer shell (**left**), internal FRD334 structure (**center**), and their combined configuration shown as a cross-section (**right**). (**c**) Proposed design–simulation–manufacturing loop for a shape-changing boat fendering system. Reproduced from ref. [134] (**a**), ref. [15] (**b**), and ref. [123] (**c**).

The unique capabilities of 4D-printed energy absorbers enable transformative applications across multiple sectors, as shown in Figure 13. In the automotive industry, reversible and programmable structures improve crashworthiness while reducing repair costs and downtime [121]. Aerospace systems benefit from lightweight, multifunctional designs for landing gear and structural protection, including vibration-damping metamaterials [163]. In protective equipment, reusability and customization enhance safety and reduce lifecycle costs [15,157]. Packaging applications leverage reusable, tailored energy absorption for the sustainable protection of high-value goods [37,157]. Biomedical devices exploit shape memory behavior for deployable implants and minimally invasive applications [121,160]. Marine systems utilize reusable metamaterial fenders for impact mitigation during docking [123]. Additionally, multifunctional TPMS-based structures enable combined mechanical protection and electromagnetic shielding, supporting applications in electronics, telecommunications, and aerospace systems [138].

## 8. Limitations and Future Research Directions

### 8.1. Benchmarking and Performance Synthesis

To critically evaluate the transformative potential of additively manufactured (AM) architectures, it is imperative to benchmark their energy absorption capabilities against established industry standards. While the preceding sections detail the individual mechanical responses of various 2D, 3D, and 4D structures, synthesizing these findings provides a unified perspective on the current state of the art. In conventional impact mitigation engineering, material selection is often constrained by well-documented performance envelopes. According to foundational literature on cellular solids and energy absorption [168,169], conventional polymeric foams (CPF) typically exhibit specific energy absorption (SEA) values in the range of 1–10 J/g [170]. Traditional honeycombs (TH), widely used in structural sandwich panels, offer slightly higher capacities, generally between 5 and 20 J/g. Furthermore, conventional aluminum foams (AlF), which frequently serve as the benchmark for lightweight metallic absorbers, typically reach their functional limit at approximately 10–30 J/g [171].

**Figure 14:**
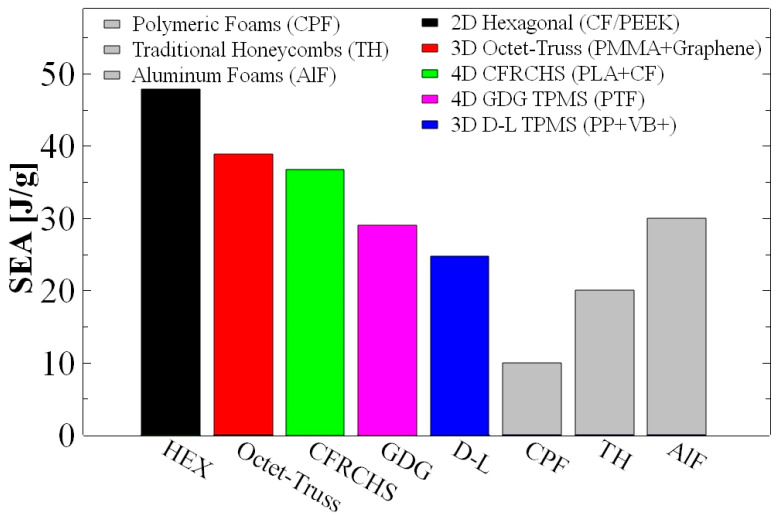
Benchmarking of the five highest specific energy absorption (SEA) values reported in the reviewed literature for additively manufactured (AM) polymeric structures against the typical performance ranges of conventional materials. Abbreviations: CPF—conventional polymeric foams; TH—traditional honeycombs; AlF—aluminum foams [168,169,170,171]. The data highlight the exceptional energy dissipation capacity of advanced AM topologies (HEX [98], octet-truss [69], CFRCHS [75], GDG [167], and D-L [129] TPMS variants) compared to conventional cellular solids. Data points are compiled from the studies discussed in Section 3, Section 4, Section 5, Section 6 and Section 7 and from foundational literature.

To illustrate how AM technologies disrupt these traditional boundaries, Figure 14 presents a comparative benchmarking of the five highest-performing SEA configurations identified in this review. In contrast to conventional materials, optimized AM topologies consistently exceed established performance thresholds. As shown, fiber-reinforced composites—such as the 2D hexagonal CF/PEEK lattice [98] and the 4D-printed CFRCHS (PLA + CF) architectures [75]—demonstrate exceptional mass-specific energy dissipation, reaching peak SEA values approaching 48 J/g and 36.75 J/g, respectively. Similarly, advanced 3D topological designs without fiber reinforcement, such as the PMMA–graphene octet-truss [69] and optimized TPMS variants (GDG [167] and D-L [129]), achieve SEA values ranging from 24 to 38.9 J/g.

This quantitative synthesis substantiates the superior mass-specific efficiency of optimized AM structures. By surpassing the ~30 J/g upper limit typical of conventional metallic foams, these polymeric and composite AM architectures demonstrate their viability as highly efficient, lightweight alternatives. The convergence of advanced material extrusion and photopolymerization techniques with complex, stress-optimized geometries indicates that AM energy absorbers are no longer merely theoretical concepts but are increasingly ready for integration into high-performance impact mitigation systems in the aerospace, automotive, and protective equipment industries.

### 8.2. Current Limitations

Despite the rapid growth of research on additively manufactured polymeric energy-absorbing architectures, several limitations hinder robust comparison and practical implementation. The literature remains highly heterogeneous in testing protocols, including loading conditions, strain-rate definitions, specimen geometry, and densification criteria, complicating cross-study benchmarking and meta-analysis [40].

Manufacturing-related variability, such as print orientation, interlayer adhesion, porosity, and process-induced defects, has a significant impact on key performance metrics (SEA, volumetric energy absorption, CLE), yet is often insufficiently quantified or controlled [26,27]. This lack of rigorous documentation has contributed to a growing reproducibility crisis in AM lattice testing. Small variations in print orientation, the use and removal of support structures, or specific post-processing conditions can significantly alter the mechanical response and failure modes, yet these critical parameters are frequently underreported or entirely omitted in the literature. Additionally, many high-performance studies rely on limited sample sizes or single-impact tests, lacking comprehensive evaluation of durability, fatigue behavior, and environmental stability (e.g., temperature, humidity, UV), which are essential for industrial and safety-critical applications [28,29].

Multi-material and continuous fiber-reinforced architectures offer enhanced performance but introduce complex interfacial failure mechanisms, including adhesion loss, delamination, and microstructural defects, which remain poorly characterized and limit reproducibility [52,105]. Furthermore, although emerging 4D and shape memory systems demonstrate promising reusability and adaptive behavior, current research is largely confined to proof-of-concept studies, with limited systematic data across loading regimes and repeated cycles [37,154].

### 8.3. Future Research Directions

To accelerate the transition from laboratory-scale studies to practical applications, coordinated research efforts are required across several key areas. Standardization and benchmarking remain critical, requiring the establishment of unified testing protocols, such as strain-rate regimes, densification criteria, and specimen geometries, alongside publicly accessible benchmark datasets to enable reliable comparison and model validation [35,40].

A deeper understanding of manufacturing–performance relationships is also needed, particularly the influence of process parameters (e.g., orientation, layer height, energy density, and post-processing) on defect formation and macroscopic performance metrics such as SEA, volumetric energy absorption, and CLE, including the effects of thermal treatments and powder aging in SLS/MJF processes [26,27,151].

Long-term durability must be systematically addressed through cyclic loading, multi-impact testing, and environmental aging studies (temperature, humidity, UV), especially for reusable elastomeric and 4D systems, where performance retention is critical [28,29,86]. In parallel, interfacial mechanics in multi-material and fiber-reinforced architectures require dedicated investigation using fracture-based methods (DCB, ENF, peel) combined with microstructural characterization to establish robust design guidelines for hybrid and graded systems [105,145].

Scalability and manufacturability remain key challenges, necessitating the development of hybrid production strategies, cost models, and in-line quality control approaches, including nondestructive evaluation and process monitoring, to support industrial deployment [30,63]. Furthermore, the expansion of open-access datasets will enable data-driven design approaches, including machine-learning-assisted topology optimization and uncertainty-aware modeling, validated through interlaboratory studies [19,141].

Sustainability considerations should also be integrated, with increased focus on recyclability, end-of-life strategies, and lifecycle environmental impacts compared to conventional materials [15,37]. Finally, emerging adaptive and 4D systems require validation at the system level, moving beyond single-component studies toward realistic application scenarios that assess recovery, durability, and energy absorption under complex loading conditions [37,154]. Furthermore, the integration of smart infills, such as phase-change materials (PCMs) or shear-thickening fluids (STFs), within 3D-printed rigid lattices represents a promising yet underexplored approach for developing next-generation adaptive energy absorbers.

Regarding end-of-life strategies, thermoplastic energy absorbers offer significant potential for circularity through mechanical recycling. Crushed or failed structures can be shredded, cleaned, and re-extruded into new filament or powder feedstock. However, repeated recycling may lead to molecular weight degradation and a slight reduction in mechanical performance, a trade-off that requires further investigation to ensure the reliability of recycled materials in impact-critical applications. Addressing these priorities will be essential to bridge the gap between experimental research and the deployment of reliable, high-performance energy-absorbing systems.

## 9. Conclusions

This review systematically analyzed over 200 additively manufactured polymeric energy-absorbing structures reported between 2016 and 2026, encompassing 2D and 3D bulk architectures, sandwich systems, and emerging 4D designs. The results highlight that structural topology, material selection, and manufacturing technique jointly govern energy absorption performance.

Clear distinctions were identified across structural classes. 2D architectures, particularly those based on high-performance polymers (PEEK, CF/PEEK), achieve the highest mass-specific energy absorption (up to 47.9 J/g), whereas 3D lattices provide superior volumetric efficiency (>5.7 J/cm^3^) due to distributed load-bearing networks. Sandwich structures offer an effective compromise, combining moderate energy absorption with high bending stiffness (up to 2950 N/mm). These findings emphasize the need to tailor architecture to application-specific requirements.

Material selection remains critical. High-performance thermoplastics maximize energy absorption but are limited by cost and processing complexity, while commodity polymers (PLA, ABS, PETG) dominate due to accessibility. Elastomers such as TPU enable reusable systems with high crush load efficiency (87–94%), and composite reinforcement further enhances performance when aligned with loading conditions.

Topological design plays a central role in controlling deformation mechanisms. TPMS structures consistently demonstrate superior performance due to smooth geometries and uniform stress distribution, while auxetic and bioinspired architectures provide enhanced damage tolerance and multi-scale energy dissipation. Concurrently, AM technologies define achievable complexity and performance, with FDM offering accessibility, SLA/DLP enabling high-resolution geometries, and SLS/MJF supporting complex, support-free architectures. Emerging hybrid approaches further expand design capabilities.

Overall, optimal energy-absorbing systems require integrated design strategies that balance topology, material behavior, and manufacturing constraints. Functionally graded, hierarchical, and multi-material architectures offer promising routes to improve efficiency and control force transmission. The significance of this comprehensive review lies in establishing a roadmap for the transition from traditional, passive energy absorbers to next-generation adaptive systems. By systematically mapping the interplay between architectural topology, fabrication variability, and mechanical performance, this study highlights the need to bridge the “standardization gap” to ensure industrial scalability and cross-study comparability. Looking forward, the field is poised for a paradigm shift driven by the convergence of 4D printing and data-driven design. The integration of shape-morphing capabilities and machine-learning-optimized metamaterials will enable the development of “smart” protective structures capable of real-time reconfiguration. Ultimately, aligning these advances with circular economy principles—through the use of bio-based materials and closed-loop recycling strategies—will be essential for the widespread commercialization of sustainable, high-performance impact mitigation solutions in the aerospace, automotive, and biomedical sectors.

## Figures and Tables

**Table 1:** Summary of processing parameters, material systems, and post-processing steps for major AM technologies used in polymeric energy absorption applications, including FDM, SLA/DLP, and SLS/MJF.

Parameter	FDM	SLA/DLP	SLS/MJF
Materials	PLA, ABS, PA, TPU, PETG, PEBA	Photopolymer resins (standard, tough, flexible)	PA12, PA11, PA-CF
Feedstock form	Filament	Liquid resin	Powder
Layer thickness	0.1–0.3 mm	25–100 μm	80–150 μm
Temperature/Energy	Nozzle: 190–270 °C; Bed: 40–110 °C	UV exposure: 1–10 s/layer	Laser power: 15–50 W
Speed	20–100 mm/s	Lift speed: process-dependent	Scan speed: 1000–5000 mm/s
Resolution	Moderate	High	Moderate–high
Supports	Required	Required	Not required
Key feature	Anisotropic	High precision, isotropic	Uniform, dense structures
Post-processing	Support removal, annealing (60–80 °C, 1–4 h)	IPA wash (5–10 min), UV cure (15–60 min)	Powder removal, optional coating
Critical factors	Orientation, layer bonding	Exposure time, support design	Energy density, powder quality

**Table 2:** Summary of representative 2D cellular structures, materials, and energy absorption performance.

Structure Type	Material	Printing Method	Testing Mode	E_abs_ (J)	SEA (J/g)	Eff. Abs. (%)	Ref.
Honeycomb	PLA	FDM	OofPC	-	20.94	-	[101]
Bamboo				-	24.8	-	
Snake-like				-	12.15	-	
RH	PP (RGD450)	Polyjet	InPC	-	4.07	-	[96]
Honeycomb	TPU	FDM	InPC	-	-	36	[53]
Honeycomb	PLA + Cont CF	Modified FDM	InPC	-	5.32	-	[95]
Chiral structure	PLA + CF	FDM	InP, tensile	-	0.05 (J/cm^3^)	-	[99]
DAP	PA	FDM	InPC	-	-	-	[102]
	PA + Short CF	FDM	InPC	-	0.58	-	
Windmill-like shape (N01)	PA12	MJF	InPC	-	1.3	-	[97]
E. aspergillum sea sponge structure	TPU	FDM	InPC	-	0.946	39.8	[100]
RCA	PA12	MJF	InPC	-	2.3	-	[103]
2RH	PA12 + Short CF	FDM	InPC	-	5.4	-	[104]
Honeycomb	PLA + TPU	FDM	InPC	-	1 (J/cm^3^)	-	[105]
Honeycomb variations	PA12 + TPC	FDM	InPC	-	-	-	[106]
Hexagonal	CF/PEEK	FDM	InPC	-	3.4	-	[98]
Chiral					4.2		
Re-entrant					4.4		
Hexagonal	PEEK				5.6		
Chiral					4.8		
Re-entrant					4.4		
Hexagonal	CF/PEEK		OofPC	-	47.9	-	
Chiral					44.7		
Re-entrant					45		
Hexagonal	PEEK				43.6		
Chiral					41		
Re-entrant					43		
Star hourglass honeycomb	PLA	FDM	InPC	-	0.024 (J/cm^3^)	-	[107]
	PLA + CF				0.062 (J/cm^3^)		
	PLA + GF				0.049 (J/cm^3^)		
Honeycomb	PA	FDM	InPC	29.84	1.08	-	[108]
	PA + Short CF			47.5	1.24	-	
	PA + GF			43.27	1.11	-	
RH	PA	FDM	InPC	41.77	1.15	-	
	PA + Short CF			95.86	1.84	-	
	PA + GF			86.79	1.65	-	
Honeycomb	ABS	FDM	OofPC	15.11	-	-	[109]
	TPU			2.91	-	-	
	ABS + TPU			7.99	-	-	
Square honeycomb	ABS	FDM	OofPC	11.5	-	-	
	TPU			2.18	-	-	
	ABS + TPU			4	-	-	
Honeycomb	ABS	FDM	InPC	1.96	-	-	
	TPU			0.64	-	-	
	ABS + TPU			1.37	-	-	
Square honeycomb	ABS	FDM	InPC	2.58	-	-	
	TPU			0.12	-	-	
	ABS + TPU			0.98	-	-	
RH	PLA	FDM	OofPC	-	6.3	-	[110]
HHIH				-	8.9	-	
HHOH				-	10.4	-	
HCOH				-	11.3	-	
Re-entrant	ABS	FDM	InPC	78	2.5	-	[111]
Arrowhead				125	3.5	-	
Anti-tetra chiral				110	3.7	-	
RH	ABS	FDM	InPC	2.95	2.82	-	[112]
RH	PLA + GF	Modified FDM	InPC	244.3	3.8	-	[19]
	PA6 + GF			48.18	0.8	-	
	PLA + GF + Foam			298	4.55	-	
	PA6 + GF + Foam			82.52	1.32	-	
Honeycomb	PET-G	FDM	InPC	66.71	3.51	-	[45]
Double-layered helix honeycomb	PLA	FDM	InPC	-	6.4	-	[113]
Graded honeycomb	TPU	FDM	InPC, impact test	-	0.026–0.119 (J/cm^3^)	-	[86]
Bone bioinspired thin-walled cellular structure	PA	FDM	InPC	97.3	12.97	-	[114]
Honeycomb				57.5	8.91	-	
RH				19.6	2.11	-	
VSC-004	PA12	FDM	InPC, impact test	-	0.297	-	[74]
Honeycomb	TPU95A	FDM	InPC	-	1.44	47	[28]

**Table 3:** Summary of representative 2D sandwich structures and their performance metrics.

Structure Type	Material	Printing Method	Testing Mode	E_abs_ (J ∗ cm^3^/g)	SEA (J/g)	Eff Abs. (%)	Ref.
InP rectangular core	PLA	FDM	Lvi	-	-	84.8	[117]
InP hexagonal core						71.4	
InP auxetic core						70.7	
OofP rectangular core						52.6	
OofP hexagonal core						51.4	
OofP auxetic core						61.75	
Honeycomb	TPU	FDM	QsC	-	0.83	-	[55]
		FDM + scCO2			1.04		
DNA molecule’s base structure (F’’)	TPU	FDM	QsC	-	0.23	-	[115]
Accordion cellular structure	PLA + CF + foam	FDM	QsC	-	1.64	-	[118]
	PLA + CF			-	1.95	-	
Auxetic core Sandwich panel	PETG	FDM	Lvi (2.5 m/s)	3.02	-	-	[56]
	PETG + CF			2.77			
Hexagonal core Sandwich panel	PETG			3.04			
	PETG + CF			2.84			
Chiral frame	PA2200	SLS	QsC	9.54	0.53	-	[120]
Foam-filled frame	PA2200 + foam			15.23	0.65	-	
Hexagonal core Sandwich panel (C3)	PP + CFRP	FDM	Impact (20 J)	-	0.1	94	[76]
RH	PLA + PU foam	FDM	3PB	-	0.2	-	[21]
	TPU + PU foam				0.195		
Hexagonal structure	PLA + PU Foam			-	0.3	-	
	TPU + PU foam				0.271		

**Table 4:** Summary of representative 3D lattice structures and their energy absorption performance.

Structure Type	Material	Printing Method	Testing Mode	E_abs_ (J)	SEA (J/g)	SEA (J/cm^3^)	Eff Abs. (%)	Ref.
DAPL	TPU	SLS	QsC, Lvi	-	-	4.476	-	[62]
TT-DAPL						4.476	89.7	
ST-DAPL						5.034	93.6	
HT-DAPL						4.332	87.3	
Octet unit cell	PA	FDM	QsC	736	13.8	-	-	[52]
	PA + CF			954	-	-	-	
	mixt PA + PA-CF			890	13.7	-	-	
Square origami honeycomb	TPU	FDM	QsC	-	-	-	49	[133]
Primitive IPC	VWP + A30	PolyJet	QsC, 3PB	-	-	1.75	-	[68]
3D star-triangular auxetic honeycomb	TPU	MJF	QsC	2.55	0.0543	-	-	[134]
3D star-triangular auxetic honeycomb	PA 12			24.1	0.616			
3D re-entrant honeycomb	PA	-	QsC	-	-	-	-	[135]
Victoria Water Lily-inspired structure	-	SLA	QsC	-	-	-	-	[60]
Octet-truss unit cells	ABS	SLA	QsC	-	-	-	45	[91]
Schwarz P	VWP + A30	PolyJet	QsC	-	-	1.15	-	[127]
Hybrid three-dimensional cubic lattice	PLA	FDM	QsC	-	0.0045	0.9	46–47	[72]
Gyroid	PA + CF	FDM	QsC	128	8.82	-	47	[136]
Diamond and Cubic	PLA/CaCO3	FDM	QsC	-	-	-	-	[137]
	PLA/TCP							
Gyroid	PLA + CNT	FDM + Dipping	QsC	-	6.91	-	-	[138]
Hybrid structure	PLA	FDM	QsC	-	-	0.663	48.32	[139]
Spherical-shell lattice structure	TPU	SLS	QsC	-	-	0.019	-	[140]
Spinodal shell	VWP + A30	Polyjet	QsC	-	-	5.76	-	[128]
Spinodal solid						5.67		
Octet lattice						4.38		
Schwarz P						4.24		
Hybrid bio-inspired lattice cellular structure	Resin	SLA	QsC	-	1.82	-	-	[141]
Cubic Truss	Resin 50A	SLA	QsC	-	0.13	-	-	[87]
Diagonal Triss					0.11			
Square Void					0.051			
Schwarz P					0.1			
Schwarz D					0.15			
SC-BCC-FCC	PlasGRAY	SLA	Lvi	-	5.02	-	-	[41]
SC					2.92			
BCC					0.9			
FCC					1.38			
Cubic cell (LS1)	ABS	FDM	QsC, Lvi	-	0.1 (static) 0.45 (impact)	-	-	[142]
Pyramidal cell (LS2)					0.43 (static) 0.85 (impact)			
PUF + LS2-S7.5	ABS + PUF				0.70 (static) 0.98 (impact)			
Circular	PA12	MJF, FDM	QsC	-	-	0.51	51	[132]
Octagonal						0.75	54	
Strengthened Octagonal						0.55	57	
Kelvin						0.59	53	
RO						0.46	47	
Cubic						0.41	27	
Cube with honeycomb infill pattern	EVA	FDM pellet	QsC	-	-	-	-	[143]
SC-BCC-FCC	PPR + MWCNT	FDM	Lvi	-	16.1	-	-	[81]
SC-BCC-FCC	HDPE + MWCNT				19.9			
Schwarz-Diamond	PLA	FDM	Ballistic test	-	0.167	0.375	-	[144]
Schwarz-Diamond	PA6	FDM	QdC	-	26.8	-	-	[145]
	sCF + PA6				26.1			
	PLA				25			
Orthotropic lattice structure	LCE (in house)	DLP	QsC	-	4.72	-	-	[61]
3D-RP-6	Tough resin	SLA	QsC	-	0.71	0.12	-	[130]
3D-ML-6	Tough resin + Ni/Ni − Cu/Ni				1.36	0.67		
Gyroid	ABS	FDM	QsC	-	10.49	-	-	[146]
	PLA				8.96			
	TPU				0.96			
	PA12				14.16			
P-cell	ZrO2 mixed photopolymer resin	DLP	QsC	-	-	0.14	-	[147]
Gyroid						0.34		
IWP						1.06		
S14						1.32		
D-L TPMS	PP+ VB+	PolyJet	QsC	-	24.75	-	-	[129]
IWP-L TPMS					23.86			
G-L TPMS					23.46			
G-S TPMS					23.21			
Spherical	ABS	FDM	QsC	-	-	-	-	[84]
Elliptical-1								
Elliptical-2								
Random sphere								
Tetrakaidecahedron								
Octet-A								
Octet-B								
Cubic-Octet-A								
Cubic-Octet-B								
DMTL 14	Clear blue photopolymer resin	SLA	QsC	-	0.31	-	-	[148]
TMTL 135					0.44			
QMTL 1245					0.46			
3DF					0.49			
Sea urchin shell	TPU	FDM	QsC	-	-	-	-	[149]
Kelvin foam structure	TPU7/PLA3	FDM	QsC	-	-	0.5	-	[150]
HGF	PA12	MJF	QsC	-	-	2.18	-	[63]
Graded TPMS P						1.655		
Octet-truss lattice structure	PMMA	SLA	Tensile, QsC	-	0.59	-	-	[151]
Octet-truss lattices	PMMA + graphene	SLA	QdC	-	38.9	-	-	[69]
NSSs	PA12	SLS	QsC	-	-	18.3 × 10^−5^	-	[131]
FRD334 (TPMS)	PEBA	FDM	QsC	-	1.22	-	-	[15]

**Table 5:** Representative 3D sandwich structures and their performance metrics.

Structure Type	Material	Printing Method	Testing Mode	E_abs_ (J)	SEA (J/g)	Bending Stiffness (N/mm)	Ref.
FGLB AAL-AL-FRP	UTR8119 + Al	SLA	3PB, impact	-	4.58	-	[85]
Gyroid AAG-AL-FRP				-	4.92	-	
Primitive AAP-AL-FRP				-	3.9	-	
NSMS	PA + GFR	SLS	QsC, impact	-	0.034 (J/cm^3^)	-	[73]
Honeycomb	TPU/PVDF	FDM + CO_2_ foaming	QsC	-	2.2	-	[119]
Six gradient stiffness structure	TPU/PVDF	FDM		-	3.3	-	
Primitive	ABS	FDM	3PB	30.2	-	1698.5	[79]
Neovius				20.7	-	1914.4	
IWP				19.1	-	1868.2	
Cubic core	PLA	FDM	3PB, Lvi	-	-	2950	[80]
Octetand core				-	-	2840	
Isomax cellular core				-	-	2900	

**Table 6:** Summary of the most representative 4D structures and their performance metrics.

Structure Type	Material	Printing Method	Testing Mode	SEA (J/g)	Ref.
ZPR Model D	Tough 1500 Resin	SLA	QsC	2.24	[160]
ZPR Model E				2.06	
ZPR Model F				2.6	
Hexagonal horseshoe shape	PLA	FDM	QsC	0.065	[157]
Square horseshoe shape				0.043	
Honeycomb 52◦	PLA + CFRCHS	FDM modif	OofPC	32.23	[75]
Honeycomb 63◦				34.12	
Honeycomb 90◦				36.75	
Honeycomb 52◦			InPC	11.4	
Honeycomb 63◦				14.2	
Honeycomb 90◦				10.13	
NPR	PLA	FDM	InPC	7.92	[94]
ZPR				6.8	
PPR				8.3	
Rhombus	PLA/TPU (70/30) + CFRLS	FDM modif	QsC	-	[46]
Auxetic 1				0.284	
Auxetic 2				0.324	
Auxetic meta-sandwich	FlexPro + SMP	FDM	QsC	-	[37]
Re-entrant auxetic structure	PLA	FDM	QsC	-	[121]
AuxHex structure				-	
Hexagonal structure				-	
Hexagonal honeycomb with PPR	PLA	FDM	QsC	-	[159]
Hybrid honeycomb with ZPR				-	
RHwith NPR				-	
Hexagonal honeycomb with PPR	PETG			-	
Hybrid honeycomb with ZPR				-	
RHwith NPR				-	
C-corrugated-1	PLA+ silk fibers	FDM	QsC	0.69	[161]
C-corrugated-2				0.73	
D-corrugated-2				0.48	
Lozenge	PLA	FDM	QsC	0.68	[122]
Tetrachiral				0.97	
Antitetrachiral				0.98	
Rototetrachiral				1.32	
Hexachiral				2.31	
Rotochiral				2.55	
Woven metamaterial (Type 1–3)	SMP PLA	FDM	QsC, 3PB	3.3	[163]
FG honeycomb II	PLA	FDM	QsC	1.13	[123]
FG re-entrant II				1.18	
FG RCA II				1.15	
GD	PTF	FDM	QsC	26	[167]
GDG				29	
DGD				23	

## Data Availability

No new data were created or analyzed in this study.

## Abbreviations

2D	Two-dimensional
2RH	Two-dimensional re-entrant honeycomb structure variant
3D	Three-dimensional
3DF	Three-dimensional lattice structure variant
3D-ML-6	3D-printed multilayer lattice structure model
3D-RP-6	3D-printed lattice structure model
3DSTH	3D star-triangular auxetic honeycomb
3PB	Three-point bending test
4D	Four-dimensional
AAG-AL-FRP	Sandwich configuration comprising a Gyroid core, aluminum face sheets, and FRP reinforcement
AAL-AL-FRP	Sandwich configuration comprising an FGLB core, aluminum face sheets, and FRP reinforcement
AAP-AL-FRP	Sandwich configuration comprising a Primitive core, aluminum face sheets, and FRP reinforcement
ABS	Acrylonitrile butadiene styrene
Al	Aluminum
AlF	Aluminum foams
AM	Additive manufacturing
ASTM	American Society for Testing and Materials
AuxHex	Auxetic hexagonal topology
BCC	Body-centered cubic lattice
CaCO_3_	Calcium carbonate
CAD	Computer-aided design
CEA	Conventional energy absorption
CF	Carbon fiber
CF/PEEK	Carbon fiber-reinforced polyetheretherketone
CFRCHS	Continuous fiber-reinforced composite honeycomb structure
CFRLS	Continuous fiber-reinforced lattice structure
CFRP	Carbon fiber-reinforced polymer
CLE	Crush load efficiency
CLIP	Continuous liquid interface production
CMM	Coordinate measuring machine
CNT	Carbon nanotubes
CPF	Conventional polymeric foams
d	Total compression displacement up to the densification point
DAP	Double arrowhead plate topology
DAPL	Double arrowhead plate-lattice architecture
DCB	Double cantilever beam
DGD	Heterogeneous 4D-printed lattice structure configuration
D-L	Diamond-lattice architecture (TPMS-based)
DLP	Digital light processing
DMA	Dynamic mechanical analysis
DMTL	Cellular/lattice structure variant
d_s_	Densification point displacement
EA/E_abs_	Energy absorption
ENF	End-notched flexure
EVA	Ethylene vinyl acetate
F’’	Cellular architecture inspired by the DNA molecule base structure
FCC	Face-centered cubic lattice
FDM	Fused deposition modeling
Fe_3_O_4_	Iron (II,III) oxide/Magnetite
FEA	Finite element analysis
FEM	Finite element method
FFF	Fused filament fabrication
FG	Functionally graded
FGLB	Functionally graded lattice beams
F-Koch	Fisher–Koch architecture (TPMS-based)
F_m_	Average compression force
F_p_/PCF	Peak crushing force
FRD	Triply periodic minimal surface base cell design (e.g., FRD222, FRD334)
FRP	Fiber-reinforced polymer
GD	Heterogeneous 4D-printed lattice structure configuration
GDG	Heterogeneous 4D-printed lattice structure configuration
GF	Glass fiber
GFR	Glass fiber reinforcement
G-L	Gyroid-lattice architecture (TPMS-based)
G-S	Gyroid-sheet architecture (TPMS-based)
h_0_	Initial structure height
h_c_	Compressed structure height
HCOH	Hexagonal honeycomb structure variant
HDPE	High-density polyethylene
HGF	Hybrid graded foam
HHIH	Hexagonal honeycomb structure variant
HHOH	Hexagonal honeycomb structure variant
h_r_	Recovered structure height (applicable to shape memory materials)
HT-DAPL	Double arrowhead plate-lattice auxetic structure variant
InP	In-plane architecture/orientation
InPC	In-plane compression
IoT	Internet of Things
IPA	Isopropyl alcohol
IPC	Interpenetrating phase composites
ISO	International Organization for Standardization
IWP	I-wrapped package topology
IWP-L	I-wrapped package lattice architecture (TPMS-based)
L/L_0_	Normalized specimen length
LCE	Liquid crystal elastomer
L_s_	Length-to-span ratio
LS1	Cubic cell
LS2	Pyramidal cell
Lvi	Low-velocity impact test
m	Mass of the specimen
MJF	Multi-jet fusion
MWCNT	Multi-walled carbon nanotubes
N01	Windmill-like cellular shape
NPR	Negative Poisson’s ratio
NSMS	Double curved-beam topology
NSS	Negative stiffness structures
OofP	Out-of-plane architecture/orientation
OofPC	Out-of-plane compression
p/ρ	Relative density of the cellular structure
PA	Polyamide/Nylon
PA11	Polyamide 11
PA12	Polyamide 12
PA	CF-Carbon fiber-reinforced polyamide
PCMs	Phase-change materials
PEA	Piecemeal energy absorption
PEBA	Polyether block amide
PEEK	Polyetheretherketone
PETG	Polyethylene terephthalate glycol
PLA	Polylactic acid
PLF	Peak loading force
PMMA	Polymethyl methacrylate
PP	Polypropylene
PPR	Polypropylene random copolymer
PPR	Positive Poisson’s ratio
PTF	Polymeric material system used in 4D printing applications
PU	Polyurethane
PUF	Polyurethane foam
PVDF	Polyvinylidene fluoride
QdC	Quasi-dynamic compression
QMTL	Cellular/lattice structure variant
QsC	Quasi-static compression
RCA	Re-entrant chiral auxetic topology
RH	Re-entrant honeycomb topology
RH	Relative humidity
RO	Three-dimensional lattice structure variant
R_r_	Shape recovery ratio
S14	Cellular/lattice structure variant
SC	Simple cubic lattice
scCO_2_	Supercritical carbon dioxide
sCF	Short carbon fiber
SEA	Specific energy absorption
SEM	Scanning electron microscopy
SLA	Stereolithography
SLM	Selective laser melting
SLS	Selective laser sintering
SMA	Shape memory alloys
SME	Shape memory effect
SMP	Shape memory polymers
ST	DAPL-Double arrowhead plate-lattice auxetic structure variant
STFs	Shear-thickening fluids
t	Structural element thickness (e.g., cell wall, strut, or plate)
TCP	Tricalcium phosphate
T_g_	Glass transition temperature
TH	Traditional honeycombs
TMTL	Cellular/lattice structure variant
TPC	Thermoplastic copolyester
TPMS	Triply periodic minimal surface
TPU	Thermoplastic polyurethane
TT-DAPL	Double arrowhead plate-lattice auxetic structure variant
UTM	Universal testing machine
UV	Ultraviolet
VWP + A30	VeroWhitePlus and Agilus 30
x	Displacement recorded during compression
Z	Nodal connectivity number
ZPR	Zero Poisson’s ratio
ZrO_2_	Zirconium dioxide
ε_0_	Maximum peak strain
ε_D_/ε_d_	Strain prior to the densification zone/Densification strain
η_abs_	Energy absorption efficiency
σ_m_	Average stress
σ_p_	Maximum stress value
σ_s_	Specific stress of the linear plateau
σ_v_	Specific stress of the maximum peak
